# High protein intake on later outcomes in preterm children: a systematic review and meta-analysis

**DOI:** 10.1038/s41390-024-03296-z

**Published:** 2024-06-10

**Authors:** Subhasish Das, Thomas McClintock, Barbara E. Cormack, Frank H. Bloomfield, Jane E. Harding, Luling Lin

**Affiliations:** 1 Liggins Institute, University of Auckland, Auckland, New Zealand; 2 Nutrition Research Division, International Centre for Diarrhoeal Diseases Research, Bangladesh, Dhaka, Bangladesh; 3 Newborn Services, Starship Child Health, Auckland, New Zealand

## Abstract

**Background:**

Appropriate protein intake is crucial for growth and development in children born preterm. We assessed the effects of high (HP) versus low protein (LP) intake on neurodevelopment, growth, and biochemical anomalies in these children.

**Methods:**

Randomised and quasi-randomised trials providing protein to children born preterm (<37 completed weeks of gestation) were searched following PRISMA guideline in three databases and four registers (PROSPERO registration CRD42022325659). Random-effects model was used for assessing the effects of HP (≥3.5 g/kg/d) vs. LP (<3.5 g/kg/d).

**Results:**

Data from forty-four studies (*n* = 5338) showed HP might slightly reduce the chance of survival without neurodisability at ≥12 months (four studies, 1109 children, relative risk [RR] 0.95 [95% CI 0.90, 1.01]; *P* = 0.13; low certainty evidence) and might increase risk of cognitive impairment at toddler age (two studies; 436 children; RR 1.36 [0.89, 2.09]; *P* = 0.16; low certainty evidence). At discharge or 36 weeks, HP intake might result in higher weight and greater head circumference z-scores. HP intake probably increased the risk of hypophosphatemia, hypercalcemia, refeeding syndrome and high blood urea, but reduced risk of hyperglycaemia.

**Conclusions:**

HP intake for children born preterm may be harmful for neonatal metabolism and later neurodisability and has few short-term benefits for growth.

**Impact statement:**

- Planned high protein intake after birth for infants born preterm might be harmful for survival, neurodisability and metabolism during infancy and did not improve growth after the neonatal period.
- Protein intake ≥3.5 g/kg/d should not be recommended for children born preterm.

## Introduction

Survival rates among children born preterm have increased steadily over time.^1,2^ However, these higher survival rates are accompanied by neurodevelopmental impairments, growth faltering, biochemical anomalies and other morbidities during infancy, early childhood and even in later life.^3^ The growth pattern of children born preterm is often characterised by low birth weight, faltering growth after birth, later rapid growth and accumulation of adiposity during adolescence.^4^ This growth pattern makes them vulnerable to biochemical disturbances in the first week after birth and cardio-metabolic diseases in later life.^5,6^ As adults, they experience higher odds of cardio-vascular, renal, metabolic and respiratory diseases than their term-born counterparts.^7,8^

Clinicians and nutrition researchers have used various nutrition interventions to promote growth and neurodevelopment and to prevent morbidity in children born preterm. Protein, both enterally and parenterally, is used in different forms and doses to support the postnatal growth and development of preterm infants. Adequate protein, provided immediately after birth, has the potential to improve growth, neurodevelopment and health by increasing protein accretion to support tissue growth and by interacting with insulin‐like growth factor-I-mediated endocrine mechanisms responsible for growth and neurodevelopment.^9,10^ However, high protein intake may result biochemical disturbances including metabolic acidosis, hyperammonaemia, elevated blood urea nitrogen and refeeding syndrome,^9,11–13^ and the optimum amount of protein for children born preterm remains uncertain. Protein intakes in children born preterm in different interventional studies have ranged from 2.25 to 4.5 g/kg/d or more.^14^ Many authors have recommended higher protein intakes of 3.5–4.5 g/kg/d for these children.^15–19^

Fenton et al. reported that high (≥3.0 g/kg/d) protein intake during the initial hospital stay of formula-fed preterm or low birth weight infants was beneficial for weight gain and nitrogen accretion without any clear risks.^20^ A systematic review of protein-supplemented human milk compared with unsupplemented human milk reported similar results.^9^ However, these studies did not specifically compare the benefits and risks of even higher (≥3.5 g/kg/d) protein intakes. The impact of high protein intake on later growth and the risk of later cardio-metabolic diseases during adolescence and adulthood is also uncertain.

We, therefore, conducted a systematic review and meta-analysis of the available evidence to elucidate whether high protein intake (≥3.5 g/kg/d) after birth resulted in better neurodevelopment and growth, increased biochemical anomalies or altered cardio-metabolic risk in later life for children born preterm.

## Methods

This study was registered in the International Prospective Register of Systematic Reviews (PROSPERO) database (registration number CRD42022325659). We conducted this review following the Cochrane Handbook for Systematic Reviews of Interventions.^21^ and reported it in accordance with the Preferred Reporting Items for Systematic Reviews and Meta-Analyses (PRISMA) guideline (Table S1).

### Search strategy

We searched Ovid MEDLINE, EMBASE, The Cochrane Library (the Cochrane Database of Systematic Reviews and the Cochrane Central Register of Controlled Trials (CENTRAL)), Current Controlled Trials (www.controlled-trials.com), ClinicalTrials (www.ClinicalTrials.gov), Australian and New Zealand Clinical Trials Registry (https://www.anzctr.org.au/) and WHO International Clinical Trial Registry Platform (https://www.who.int/clinical-trials-registry-platform) for relevant articles and protocols. The search strategy details are shown in Table S2. Conference abstracts were included if they provided usable summary data. No language restrictions were applied.

### Inclusion and exclusion criteria

Randomised controlled trials (RCTs) and quasi-RCTs were eligible if they compared planned high (≥3.5 g/kg/d) and low (<3.5 g/kg/d) protein intake to children born preterm (<37 completed weeks of gestation) by enteral, parenteral or both routes during the first 4 weeks after birth for a minimum duration of 5 days with the aim of improving growth or neurodevelopment or preventing morbidities or biochemical abnormalities.

### Outcome variables

The primary outcome was survival without neurodisability at or beyond 12 months’ corrected age. Neurodisability was defined as cognitive, language, and motor impairment, defined as scores 1 or more standard deviations (SDs) below the mean on standard tests of neurodevelopment or as defined by the study investigators. Secondary outcomes were survival to discharge and follow-up, neurodisability, neonatal morbidities, growth, biochemical abnormalities and cardio-metabolic outcomes. Outcomes were evaluated in infancy (≤1 year), the toddler period (1 to ≤3 years), childhood (3 to ≤8 years), adolescence (>8 to ≤18 years) and adulthood (>18 years) when data was available (Table S3).

### Screening and data extraction

We included articles from database inception to 18 January 2023. Titles and abstracts were screened using Covidence systematic review software (Veritas Health Innovation, Melbourne, Australia; www.covidence.org) by two reviewers (SD and BC) and full text of potentially relevant articles was screened by at least two of the three reviewers (SD, BC and LL). Discrepancies were resolved by discussion or with a fourth author (JH). The reference lists of the included articles were manually checked for additional articles. Data from eligible articles were extracted by two of the four reviewers (SD, BC, LL and TM) using a template and uploaded to RevMan 5.4.1 for analysis. In case of multiple reports from same trial, we used the source with most complete data. We combined means and SDs of multi-arm studies into a single group.^22^ Medians with inter-quartile ranges were converted to means and SDs.^23,24^ Protein content of breast milk was taken to be that reported in the manuscript, or 1.27 g/100 when no specific values were reported.^25^ Data from graphs were extracted using WebPlotDigitizer (https://automeris.io/WebPlotDigitizer/).

### Statistical analyses

Meta-analyses were undertaken in RevMan 5.4.1. We used random effects models for pooling the results and generated relative risks (RR) and mean differences (MD) with 95% confidence intervals (95% CI). A *p* < 0.05 was regarded as statistically significant. Heterogeneity was assessed using *χ*² tests and *I*² statistics, with *I*² > 50% and χ² *p* < 0.10 regarded as indicating significant heterogeneity. Subgroup analyses were planned to explore whether the effects of high protein intake differed with gestational age (<28 weeks, ≥28 weeks to <32 weeks, ≥32 weeks to <37 weeks), birthweight (<1000 g, ≥1000 to <1500 g, ≥1500 g to <2500 g and ≥2500 g), appropriate- versus small-for-gestational-age (birthweight < 10th percentile or as defined by investigators), timing of commencement of high protein intake (first week versus later), route of provision of high protein (parenteral, enteral or both), year of starting study (before versus after the median start date of included trials) and actual intake ≥3.5 g/kg/d versus actual intake <3.5 g/kg/d.

### Reporting

We have narrated the results of the meta-analyses and reported the conclusions based on the effect estimates from the meta-analyses and the certainty of the evidence according to the Cochrane Handbook for Systematic Reviews of Interventions version 6.3.^21^ This avoids dichotomising study results into ‘statistically significant’ and ‘not significant’ that reduces information, obscures biases and can lead to misinterpreting strong associations as null and nearly identical results as conflicting.^26^ Instead, we have followed recommendation best practice to present the quantitative interpretation of results by providing information about the magnitude and precision of effects.^26^ We categorised the certainty of evidence as high, moderate, low and very low. When the evidence was of high certainty, the effect is reported as the intervention *resulted in* a large, moderate, slight, or little or no decrease or increase in the outcome. When the evidence was of moderate certainty, the effect is reported as the intervention *likely or probably* resulted in a large, moderate, slight, or little or no decrease or increase in the outcome. When the evidence was of low certainty, the effect is reported as the intervention *might* have resulted in a large, moderate, slight, or little or no decrease or increase in the outcome. Any effect with very low certainty of the evidence is reported as little or no difference or effect on the outcome.

### Sensitivity analyses

Sensitivity analyses were undertaken excluding trials of low quality based on the Grading of Recommendations Assessment, Development and Evaluation (GRADE) outcomes, including only trials considered to have a low risk of bias for selection and detection bias and including only trials that achieved the planned high protein intake ≥3.5 g/kg/d.

### Risk of bias evaluation

Two authors independently assessed the risk of bias for each study using Cochrane risk-of-bias tool (ROB)-I for RCTs. GRADE was used to assess the certainty of evidence for the following outcomes: survival without neurodisability at or beyond 12 months’ corrected age (primary outcome); survival to discharge; cognitive impairment or delay; motor impairment or delay; presence of cerebral palsy; length/height at follow-up, and fat-free mass at follow-up. We also assessed the risk of bias at the outcome level using ROB-2 for the GRADE outcomes.

## Results

**Fig. 1:**
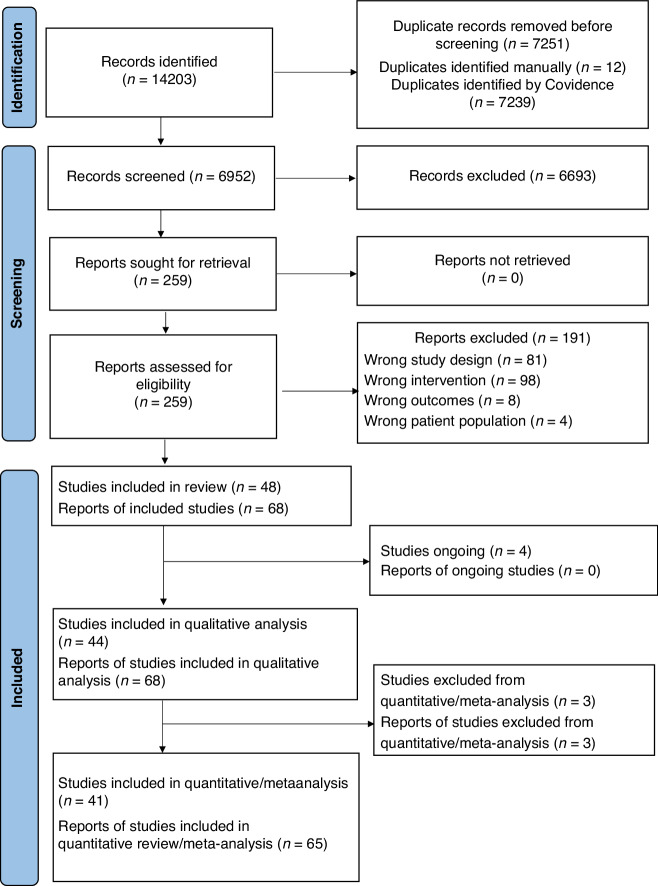
Flow diagram of study selection. Flow chart showing the literature identification and selection via databases and registers.

We identified 14,203 records through database search and 6,952 reports were left after removing duplicates. After title and abstract screening, 259 reports were sought for retrieval. We excluded 191 reports not meeting our inclusion criteria. We included data from the remaining 68 reports from 44 studies (42 RCTs and 2 quasi-RCTs) with 5,338 children born preterm (high protein, HP: 2344, low protein, LP: 2994) in the qualitative analysis. Data from 65 reports from 41 studies with 4791 children born preterm (HP: 2031, LP: 2,760) were included in the quantitative analysis (Fig. 1). Most of the studies (28 out of 32 studies that reported actual intake) achieved mean actual protein intake of ≥3.5 g/kg/d, although often not until late in the intervention period and with considerable overlap in intakes between groups. Interventions included fortified human milk, fortified bovine milk, preterm formula and amino acid solutions, given via enteral (24 studies), parenteral (14 studies), or both routes (6 studies), starting during the first week (29 studies) or after the first week after birth (8 studies) or unspecified (7 studies). The studies were conducted between 1963 and 2017 in the United States (17 studies), United Kingdom (8 studies), Italy (3 studies), Turkey (3 studies), two studies in each of India, The Netherlands and Sweden, and one study in each of New Zealand and Australia, China, Finland, Hungary, Norway, Spain and Vietnam (Table S4).

### Primary outcome

**Fig. 2:**
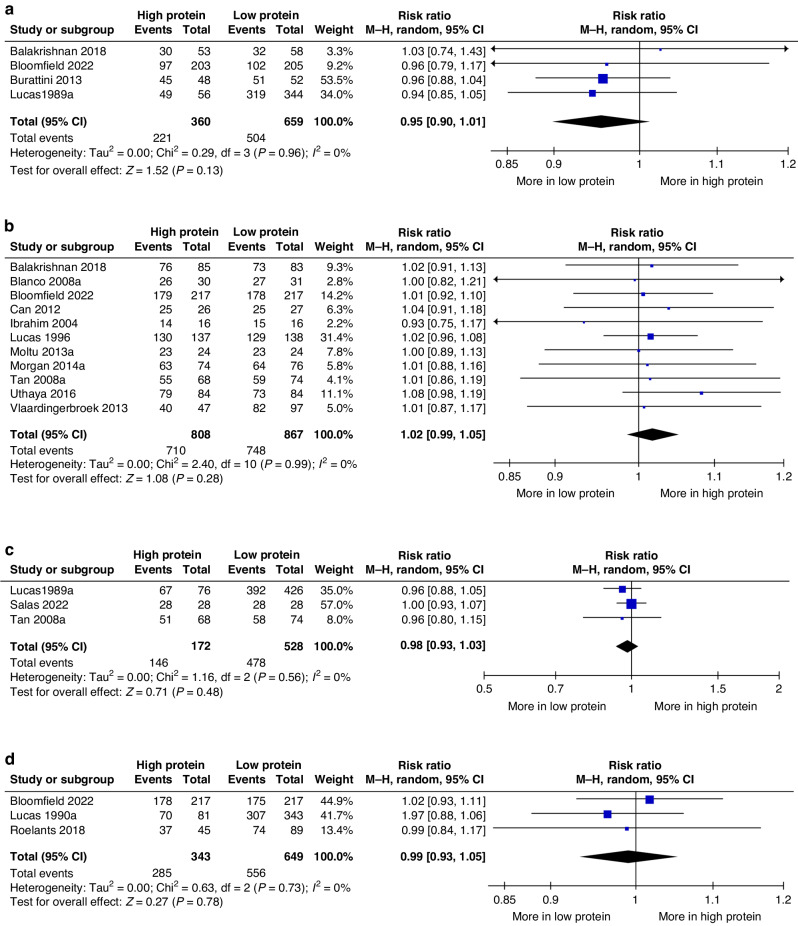
Forest plots of effects of planned high vs. low protein intake. Forest plots presenting the effects of planned high vs. low protein intake on **a** survival without neurodisability at or beyond 12 months, **b** survival to discharge or till 36–40 weeks, **c** survival up to infancy, and **d** survival up to the toddler period. CI confidence interval; M-H Mantel–Haenszel.

Meta‐analysis of data from four studies.^27–30^ showed that planned HP intake compared with LP intake might have slightly reduced chance of survival without neurodisability at or beyond 12 months’ corrected age (RR 0.95, 95% CI 0.90, 1.01; 1,019 participants; *P* = 0.13; *I*^2^ 0%; Fig. 2a) with low certainty of evidence.

### Secondary outcomes

#### Survival

Meta‐analysis of data from 11 studies^27,28,31–39^ showed that HP intake compared with LP intake had little or no effect on survival to discharge or to 36–40 weeks (RR 1.02, 95% CI 0.99, 1.05; 1675 participants; *P* = 0.28; *I*^2^ 0%; Fig. 2b) with moderate certainty of evidence. The funnel plot did not suggest significant bias due to small study effects (Supplementary File 1; Fig. A). HP intake also had little or no effect on survival to infancy (three studies.^30,37,40^; RR 0.98, 95% CI 0.93, 1.03; 700 participants; *P* = 0.48; *I*^2^ 0%; Fig. 2c) or to the toddler period (three studies.^28,41,42^; RR 0.99, 95% CI 0.93, 1.05; 992 participants; *P* = 0.78; *I*^2^ 0%; Fig. 2d)

#### Neurodisability

**Fig. 3:**
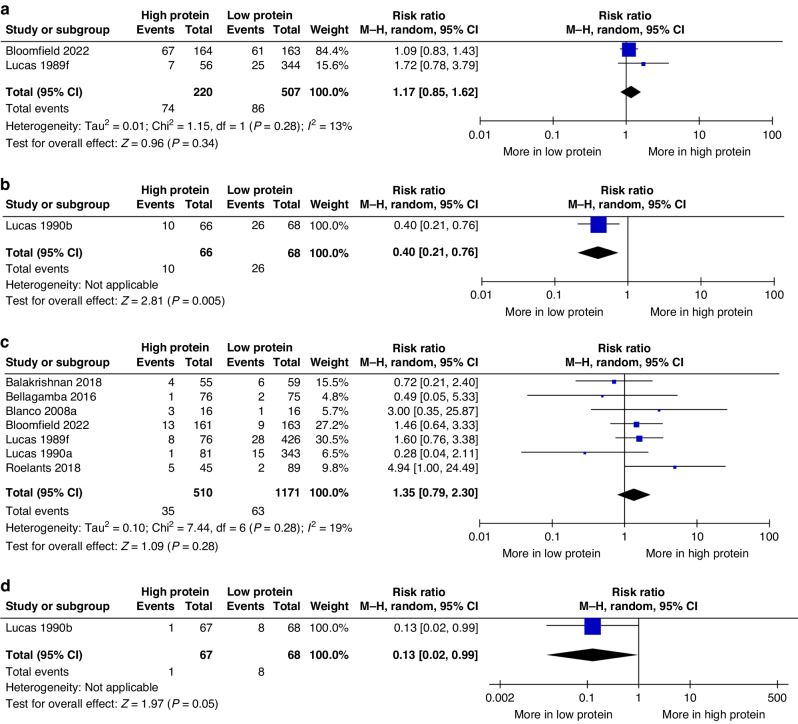
Forest plots of effects of planned high vs. low protein intake. Forest plots presenting the effects of planned high vs. low protein intake on **a** neurodisability during the toddler period, **b** neurodisability in childhood, **c** cerebral palsy during the toddler period, and **d** cerebral palsy in childhood. CI confidence interval, M-H Mantel–Haenszel.

There was little or no difference between HP and LP groups during the toddler period for neurodisability.^28,43^ (Fig. 3a), or cerebral palsy.^10,27,28,31,41–43^ (Fig. 3c). However, in childhood, one study.^44^ reported that children in the HP group had reduced risk of neurodisability (134 children; RR 0.40, 95% CI 0.21, 0.76; *P* = 0.005; Fig. 3b) and cerebral palsy (135 children; RR 0.13, 95% CI 0.02, 0.99; *P* = 0.05; Fig. 3d).

**Fig. 4:**
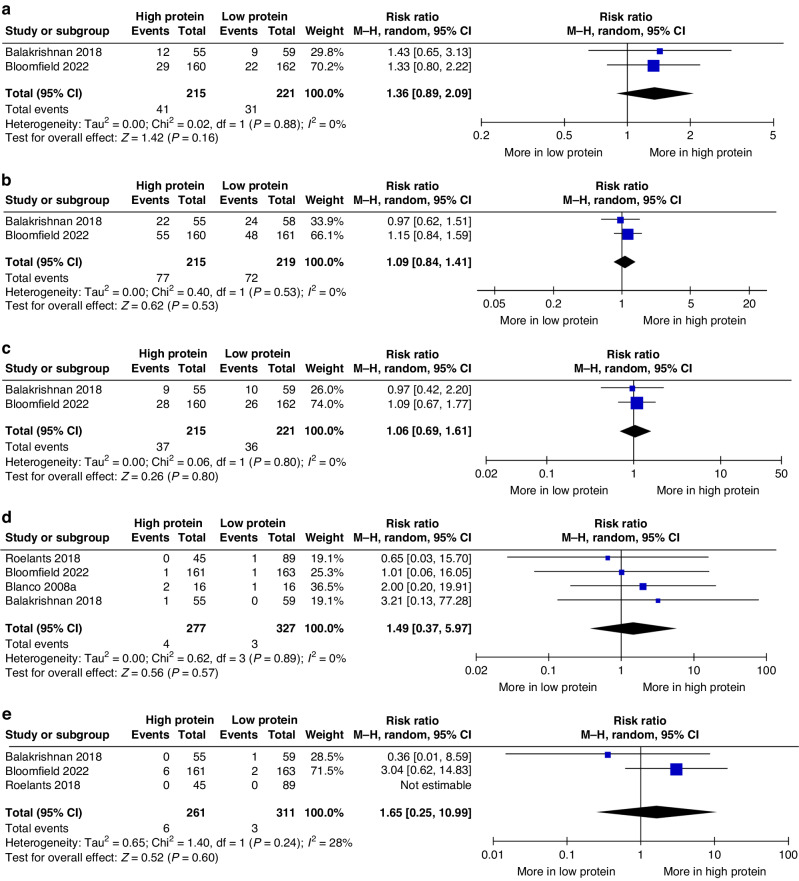
Forest plots of effects of planned high vs. low protein intake. Forest plots presenting the effects of planned high vs. low protein intake on **a** cognitive impairment or delay, **b** language impairment or delay, **c** motor impairment or delay, and **d** blindness, and edeafness during the toddler period. CI confidence interval, M-H Mantel–Haenszel.

Meta‐analysis of data from two studies found that HP intake might have increased the risk of cognitive impairment or delay during the toddler period (two studies.^27,28^; 436 children; RR 1.36, 95% CI 0.89, 2.09; *P* = 0.16; *I*^2^ 0%; low certainty evidence; Fig. 4a), but there was little or no difference in language impairment or delay.^27,28^ (Fig. 4b), motor impairment or delay.^27,28^; (Fig. 4c), blindness.^27,28,31,41^ (Fig. 4d) and deafness.^27,28,41^ (Fig. 4e).

There was little or no difference between HP and LP groups in cognitive scores in infancy.^45^ (Supplementary Fig. 1a), or during the toddler period.^10,27,28,31,34,41–43^ (Supplementary Fig. 1b). There was also little or no difference in motor scores in infancy.^45^ (Supplementary Fig. 1c) or the toddler period.^10,27,28,31,34,41–43^ (Supplementary Fig. 1d).

#### Neonatal Morbidity

There were no clear differences between high and low protein intake groups in intraventricular haemorrhage, bronchopulmonary dysplasia, retinopathy of prematurity, necrotising enterocolitis, late-onset sepsis, or patent ductus arteriosus in infancy (Table 1). Funnel plots (Supplementary File 1; Figs B to F) did not suggest significant bias due to small study effects except for retinopathy of prematurity (Supplementary File 1; Fig D).

**Table 1:** Summary of effects of planned high vs. low protein intake on neonatal morbidity.

Outcome	Studies (Participants)	Risk Ratio with 95% CI (M-H, Random)	P for overall effect	*I*^2^
Intraventricular haemorrhage	17^10,27–29,31–36,39,41,48,53,59,62^ (2225; HP:1065, LP:1160)	1.13 (0.91, 1.41)	0.27	0%
Bronchopulmonary dysplasia	18^10,27–29,31–33,35,36,39,47,53,54,59,62,66,68^ (2013; HP:968, LP:1045)	1.05 (0.96, 1.16)	0.28	0%
Retinopathy of prematurity	16^10,27–29,31–33,35,36,39,41,47,53,59,62,101^ (1904; HP:931, LP:973)	0.98 (0.76, 1.25)	0.85	0%
Necrotising enterocolitis	20^27,28,31,32,34–37,39–41,47,48,53,54,59,62,64,66,68^ (2464; HP:1170, LP:1294)	1.02 (0.77, 1.36)	0.87	0%
Late-onset sepsis	9^10,28,35,36,39,41,47,48,59^ (1243; HP:598, LP:645)	1.17 (0.95, 1.45)	0.14	29%
Patent ductus arteriosus	11^10,27–29,32,33,35,36,39,47,59^ (1484; HP:716, LP:768)	1.08 (0.93, 1.25)	0.29	13%

*HP* high protein, *LP* low protein, *CI* confidence interval, *M-H* Mantel–Haenszel test.

#### Growth

##### Weight

There was little or no difference between HP and LP groups in weight at discharge or 36 weeks.^10,27–29,37,38,40,46–54^ (Supplementary Fig. 2a), during infancy.^34,55–57^ (Supplementary Fig. 2b), during the toddler period.^10,28,29,34,55^ (Supplementary Fig. 2c) or during childhood.^55^ (Supplementary Fig. 2d). The funnel plot suggested significant bias due to small study effects for weight at discharge or 36 weeks (Supplementary file 1; Fig. G).

At discharge or 36 weeks the HP group might have slightly higher weight-for-age z-scores (11 studies.^10,27–29,36,37,40,41,49,51,52^; 1,361 children; MD 0.13, 95% CI−0.03, 0.28; *P* = 0.11; *I*^2^ 57%; Supplementary Fig. 3a), but there was little or no difference between HP and LP groups in weight-for-age z-score during infancy.^45,57,58^ (Supplementary Fig. 3b), or during the toddler period.^10,28,29,41,45,52^ (Supplementary Fig. 3c). Similarly, there was little or no difference in gain in weight z-score from birth till discharge or 36 weeks.^28,39–41,51,52,59^ (Supplementary Fig. 3d) between HP and LP groups. The funnel plot for weight z-score at discharge or 36 weeks suggested significant bias due to small study effects (Supplementary file 1; Fig. H).

##### Length

There was little or no difference between HP and LP groups in length at discharge or at 36 weeks.^10,27–29,37,38,46,47,49,51–54,60,61^ (Supplementary Fig. 4a), during infancy.^34,55–57^ (Supplementary Fig. 4b), during the toddler period.^10,28,29,34,55^ (Supplementary Fig. 4c), or during childhood.^55^ (Supplementary Fig. 4d). Similarly, there was little or no difference between HP and LP groups in length-for-age z-score at discharge or at 36 weeks.^10,27–29,37,40,49,51,52^ (Supplementary Fig. 5a), during infancy.^45,57,58^; (Supplementary Fig. 5b), or during the toddler period.^10,28,29,45,58^ (Supplementary Fig. 5c), or in gain in length z-score to discharge or 36 weeks.^28,51,52,59^ (Supplementary Fig. 5d). The funnel plot for length at discharge or 36 weeks suggested no significant bias due to small study effects (Supplementary File 1; Fig. I).

##### Head circumference

There was little or no difference between HP and LP groups in head circumference at discharge or 36 weeks. ^10,27–29,36–38,47,49–54,61^ (Supplementary Fig. 6a) or during infancy.^34,55–57^ (Supplementary Fig. 6b). Children in the HP group compared to the LP group probably had slightly smaller head circumferences during the toddler period (five studies.^10,28,29,55^; 1341 children; MD −0.33 cm, 95% CI −0.54, −0.12 cm; P = 0.002; *I*^2^ 0%; Supplementary Fig. 6c) and in childhood (two studies.^55^; 779 children; MD −0.36 cm, 95% CI −0.65, −0.07 cm; P = 0.02; *I*^2^ 0%; Supplementary Fig. 6d). However, children in the HP group might have gained slightly greater head circumference from birth until discharge or 36 weeks (12 studies.^34,39,47,48,60–67^; 1,191 children; MD 0.05 cm, 95% CI 0.01, 0.09 cm; *P* = 0.01; *I*^2^ 90%; Supplementary Fig. 6e). Funnel plots suggested no significant bias due to small study effects for head circumference at discharge or 36 weeks (Supplementary file 1; Fig. J) but significant bias due to small study effects for gain in head circumference at discharge or 36 weeks (Supplementary file 1; Fig K).

There was also little or no difference between HP and LP groups in head circumference z-score at discharge or 36 weeks.^10,27–29,36,37,40,49,52^ (Supplementary Fig. 7a). However, HP group might have smaller head circumference z-scores during infancy (three studies^45,57,58^; 150 children; MD -0.42, 95% CI -1.23, 0.39; P = 0.31; I^2^ 73%; Supplementary Fig. 7b) and the toddler period (four studies^10,28,29,45^; 601 children; MD -0.27, 95% CI −0.55, 0.01; P = 0.06; I^2^ 63%; Supplementary Fig. 7c). However, children in the HP group might have gained slightly greater head circumference z-scores from birth until discharge or 36 weeks (six studies.^28,36,39,40,52,59^; 814 children; MD 0.38, 95% CI −0.03, 0.80; *P* = 0.07; *I*^2^ 86%; Supplementary Fig. 7d).

##### Body mass index and skinfold thickness

At discharge or 36 weeks the HP group might have higher fat mass z-score (one study.^40^; 46 children; MD 1.00, 95% CI −0.01, 2.01; *P* = 0.05), fat-free mass (four studies.^38,40,49,51^; 285 children; MD 95.3 g, 95% CI −1.21, 191.7 g; *P* = 0.05; *I*^2^ 0%) and fat-free mass z-score (one study.^40^; 46 children; MD 0.60, 95% CI −0.04, 1.24; *P* = 0.07). There was little or no difference between HP and LP groups for body mass index and triceps skinfold thickness at discharge or 36 weeks, in infancy, during the toddler period or in childhood (Table 2).

**Table 2:** Summary of effects of planned high vs. low protein intake on body mass index, fat and fat free mass and skinfold thickness.

Outcome	Studies (Participants)	Mean difference with 95% CI (M-H, Random)	*P* for overall effect	*I*^2^
BMI at discharge or at 36 weeks (kg/m^2^)	1^49^ (38; HP:32, LP: 6)	−0.25 (−1.00, 0.50)	0.52	
BMI in infancy (kg/m^2^)	1^55^ (369; HP:48, LP:321)	0.12 (−0.39, 0.63)	0.64	
BMI during the toddler period (kg/m^2^)	2^55^ (772; HP:125, LP:647)	0.14 (−0.26, 0.53)	0.22	35%
BMI in childhood (kg/m^2^)	2^55^ (779; HP:129, LP:650)	−0.12 (−0.48, 0.23)	0.49	0%
Fat mass at discharge or 36 weeks (g)	4^38,40,49,51^ (285; HP: 169, LP: 116)	25.64 (−11.61, 62.90)	0.18	7%
Fat mass z-score at discharge or 36 weeks	1^40^ (46; HP:21, LP:25)	1.00 (−0.01, 2.01)	0.05	
Fat mass in infancy (g)	1^51^ (58; HP: 37, LP: 21)	70.97 (−167.92, 309.86)	0.56	
Fat-free mass at discharge or 36 weeks (g)	4^38,40,49,51^ (285; HP: 169, LP: 116)	95.3 (−1.21, 191.68)	0.05	0%
Fat-free mass z-score at discharge or 36 weeks	1^40^ (46; HP: 21, LP: 25)	0.60 (−0.04, 1.24)	0.07	
Fat-free mass in infancy (g)	1^51^ (58; HP: 37, LP: 21)	−16.70 (−253.96, 220.56)	0.89	
TSF in infancy (mm)	1^55^ (369; HP: 48, LP: 321)	0.22 (−0.27, 0.71)	0.38	
TSF during the toddler period (mm)	2^55^ (772; HP: 125, LP: 647)	0.01 (−0.36, 0.37)	0.97	2%
TSF in childhood (mm)	2^55^ (779; HP: 129, LP: 650)	−0.09 (−0.73, 0.56)	0.79	0%

*BMI* body-mass index, *HP* high protein, *LP* low protein, *CI* confidence interval, *M-H* Mantel–Haenszel test, *TSF* Triceps skin-fold thickness.

### Biochemical outcomes during infancy

Children in the HP group compared with those in the LP group probably had increased risk of developing hypophosphatemia (four studies.^28,34,35,38^; 710 children; RR 1.43, 95% CI 1.06, 1.93; *P* = 0.02; *I*^2^ 46%; Supplementary Fig. 8a), and might have increased risk of hypercalcemia (three studies.^28,34,38^; 748 children; RR 1.56, 95% CI 0.97, 2.50; *P* = 0.07; *I*^2^ 21%; Supplementary Fig. 8b), refeeding syndrome (one study.^28^; 338 children; RR 1.56, 95% CI 1.00, 2.42; *P* = 0.05; Supplementary Fig. 8c) and high blood urea concentration (one study.^34^; 275 children; RR 2.22, 95% CI 1.09, 4.50; *P* = 0.03; Supplementary Fig. 8d), but probably had a reduced risk of hyperglycaemia (four studies.^29,38,54,68^; 483 children; RR 0.61, 95% CI 0.41, 0.92; *P* = 0.02; *I*^2^ 33%; Supplementary Fig. 8e). There was little or no difference in the risk of hypoglycemia between HP and LP groups.^38^ (Supplementary Fig. 8f). Children in the HP group had slightly higher serum albumin concentrations than the LP group (six studies.^62,63,65,69–71^; 334 children; MD 0.25 g/dl, 95% CI 0.10, 0.40 g/dL; *p* = 0.001; *I*^2^ 77%; Supplementary Fig. 8g) and reduced fasting blood glucose concentrations (five studies.^27,33,39,68,72^; 460 children; MD −9.01 mg/dL, 95% CI −16.91, −1.12 mg/dL; *P* = 0.03; *I*^2^ 78%; Supplementary Fig. 9a).

### Cardio-metabolic outcomes

There was little or no difference between HP and LP groups in triglyceride concentrations in infancy.^33,39,49^ (Supplementary Fig. 9b), or in systolic.^73^ (Supplementary Fig. 9c) or diastolic blood pressure in childhood.^73^; (Supplementary Fig. 9d).

There were no data for survival and growth beyond childhood, cardiac size and structure and measures of brain growth and maturation.

### Subgroup analyses

#### Route of the intervention

There were no clear differences between HP and LP groups who received the intervention by different routes for survival without neurodisability at or beyond 12 months’ corrected age, survival to discharge, cognitive impairment at or beyond 12 months’ corrected age, motor impairment at or beyond 12 months’ corrected age, presence of cerebral palsy and length in infancy. The HP group had lower length/height during the toddler period than the LP group when they received the intervention by both parenteral and enteral routes, but the *p* value for interaction was not significant (Table 3).

**Table 3:** Summary of subgroup analyses.

Route of intervention (Parenteral, enteral, or both)						
Variable	Subgroup	Studies (participants)	RR or MD (95% CI)	*P* (overall effect)	*I*^2^	*P* for subgroup interaction
Survival without neurodisability at or beyond 12 months’ corrected age	Enteral	1 study^30^ (400; HP: 56, LP: 344)	0.94 (0.85, 1.05)	0.27	NA	0.97
	Parenteral	2 studies^27,29^ (211; HP: 75, LP: 83)	0.96 (0.89, 1.04)	0.31	0%	
	Both	1 study^28^ (408; HP: 203, LP: 205)	0.96 (0.79, 1.17)	0.69	NA	
Survival to discharge or 36–40 weeks	Enteral	1 study^34^ (275; HP: 137, LP: 138)	1.02 (0.96, 1.08)	0.62	NA	0.97
	Parenteral	7 studies^27,31–33,36,38,39^ (776; HP: 362, LP: 414)	1.03 (0.98, 1.08)	0.27	0%	
	Both	3 studies^28,35,37^ (624; HP: 309, LP: 315)	1.01 (0.94, 1.07)	0.87	0%	
Cognitive impairment or delay during the toddler period	Enteral	None				0.88
	Parenteral	1 study^27^ (114; HP: 55, LP: 59)	1.43 (0.65, 3.13)	0.37		
	Both	1 study^28^ (322; HP: 160, LP: 162)	1.33 (0.80, 2.22)	0.27		
Motor impairment or delay during the toddler period	Enteral	None				0.80
	Parenteral	1 study^27^ (114; HP: 55, LP: 59)	0.97 (0.42, 2.20)	0.93		
	Both	1 study^28^ (322; HP: 160, LP: 162)	1.09 (0.67, 1.77)	0.73		
Presence of cerebral palsy during the toddler period	Enteral	2 studies^42,43^ (926; HP: 157, LP: 769)	0.85 (0.15, 4.79)	0.85	64%	0.75
	Parenteral	3 studies^27,31,41^ (280; HP: 116, LP: 164)	1.92 (0.52, 7.08)	0.33	49%	
	Both	2 studies^10,28^ (475; HP: 237, LP: 238)	1.30 (0.60, 2.83)	0.50	0%	
Length in infancy (cm)	Enteral	2 studies^37,56^ (617; HP: 173, LP: 444)	0.18 (−0.61, 0.97)	0.66	0%	0.32
	Parenteral	None				
	Both	2 studies^34,55^ (111; HP: 56, LP: 55)	−0.75 (−2.38, 0.88)	0.37	0%	
Length/height during the toddler period (cm)	Enteral	3 studies^34,55^ (1017; HP: 250, LP: 767)	0.22 (−0.35, 0.79)	0.45	3%	0.12
	Parenteral	1 study^29^ (100; HP: 48, LP: 52)	−0.10 (−1.81, 1.61)	0.91		
	Both	2 studies^10,28^ (476; HP: 237, LP: 239)	−0.73 (−1.43, −0.03)	0.04	0%	
Actual intake ≥3.5 g/kg/d versus actual intake <3.5 g/kg/d						
Survival without neurodisability at or beyond 12 months’ corrected age	Did not achieve the ≥3.5 g/kg/d intake	1 study^29^ (100; HP: 48, LP: 52)	0.96 (0.88, 1.04)	0.28		0.95
	Achieved the ≥3.5 g/kg/d intake	3 studies^27,28,30^ (919; HP: 312, LP: 607)	0.95 (0.87, 1.04)	0.28	0%	
Length/height during the toddler period (cm)	Did not achieve the ≥3.5 g/kg/d intake	1 study^29^ (100; HP: 48, LP: 52)	−0.10 (−1.81, 1.61)	0.91		0.98
	Achieved the ≥3.5 g/kg/d intake	5 studies^34,55^ [2, 4] (1493; HP: 487, LP: 1006)	−0.13 (−0.69, 0.43)	0.66	38%	
Timing of start of study (before versus at or after median year 2008)						
Survival without neurodisability at or beyond 12 months’ corrected age	Before 2008	2 studies^29,30^ (500; HP: 104, LP: 396)	0.95 (0.89, 1.01)	0.13	0%	0.77
	At or after 2008	2 studies^27,28^ (519; HP: 256, LP: 263)	0.98 (0.82, 1.16)	0.79	0%	
Survival to discharge or 36–40 weeks	Before 2008	2 studies^33,34^ (307; HP: 153, LP: 154)	1.01 (0.95, 1.07)	0.74	0%	0.84
	At or after 2008	9 studies^27,28,31,32,35–39^(1368; HP: 655, LP: 713)	1.02 (0.98, 1.06)	0.28	0%	
Length in infancy (cm)	Before 2008	3 studies^34,37,55^ (698; HP: 212, LP: 486)	0.07 (−0.69, 0.83)	0.86	0%	0.61
	At or after 2008	1 study^56^ (30; HP: 17, LP: 13)	−0.50 (−2.56, 1.56)	0.63	0%	
Length/height during the toddler period (cm)	Before 2008	4 studies^29,34,55^ (1117; HP: 298, LP: 819)	0.18 (−0.35, 0.72)	0.50	0%	0.04
	At or after 2008	2 studies^10,28^ (476; HP: 237, LP: 239)	−0.73 (−1.43, −0.03)	0.04	0%	

*HP* high protein, *LP* low protein, *CI* confidence interval, *RR* risk ratio, *MD* mean difference, *NA* not applicable.

#### Actual intake ≥ 3.5 g/kg/d versus actual intake < 3.5 g/kg/d

There were no clear differences between HP and LP groups in survival without neurodisability at or beyond 12 months’ corrected age and length/height during the toddler period in studies that achieved planned intake ≥3.5 g/kg/d versus studies that did not achieve the planned intake ≥3.5 g/kg/d (Table 3).

#### Timing of start of study (before versus at or after median year 2008)

There were no clear differences between HP and LP groups in studies that started before versus at or after the median year (2008) for survival without neurodisability at or beyond 12 months’ corrected age, survival to discharge and length in infancy. However, the HP groups had slightly reduced length/height during the toddler period only when the study started at or after 2008 (two studies.^10,28^; 476 children; HP: 237, LP: 239; MD: −0.73 cm; 95% CI: −1.43, −0.03 cm; *P* = 0.04 for interaction) (Table 3). Other preplanned subgroup analyses were not possible due to insufficient data.

#### Studies not included in quantitative synthesis

Curran et al.^74^ reported that children in the HP group (*n* = 20) gained more weight from birth than the LP group (*n* = 38) (mean±SD, HP: 23 ± 4.9 g/d; LP:17.5 ± 4.1 g/d). Davidson et al.^75^ reported no difference in mean daily weight gains between children receiving HP (*n* = 240) and LP (*n* = 148) and Kashyap et al.^76^ reported greater weight and head circumference at age 28 days in children receiving HP (*n* = 53) compared to LP (*n* = 48), but neither study provided quantitative data for these findings.

### Sensitivity analyses

Sensitivity analyses including only trials that achieved the planned high protein intake ≥3.5 g/kg/d and including only trials considered to have a low risk of selection bias found that planned HP intake compared with LP intake might have slightly reduced chance of survival without neurodisability at or beyond 12 months corrected age (three studies.^27,28,30^; 919 participants; RR 0.95, 95% CI 0.87, 1.04; *P* = 0.28; *I*^2^ 0%; Supplementary Figs. 10a and 11a). Sensitivity analyses including only trials considered to have a low risk of selection bias found that planned HP intake compared with LP intake might have increased the risk of cognitive impairment or delay during the toddler period (one study; 436 children; RR 1.36, 95% CI 0.89, 2.09; *P* = 0.16; *I*^2^ 0%; Supplementary Fig. 11c). There was little or no difference between high and low protein intake groups for the remaining outcomes in sensitivity analyses, including only trials that achieved the planned intake and including only trials considered to have low risk of selection and detection bias (Supplementary Figs. 10, 11, 12), but in all the cases, the direction and size of the effect estimates were similar to those of the overall analyses.

### Risk of bias assessment

Fourteen studies had a high risk of performance bias due to the lack of blinding of participants and personnel (Supplementary Fig. 13). Additionally, ten studies had high risk of detection bias due to the lack of blinding of outcome assessors, and ten had selection bias due to lack of allocation concealment. The high risk of other bias in several studies was due to attrition bias resulting from incomplete outcome data (eight studies), selective reporting bias (six studies), and selection bias due to lack of random sequence generation (four studies). For GRADE outcomes assessed using ROB2, there were low to some concerns of risk of bias for all outcomes except for high risk of bias for the primary outcome due to missing data.^29^ and for length in infancy due to bias in selection of reported results.^35^ (Supplementary Fig. 14).

### Certainty of evidence (GRADE)

The certainty of the evidence was assessed as moderate to low or very low for all GRADE outcomes (Table 4).

**Table 4:** GRADE table: Summary of findings.

Outcome	No of studies (total participants)	Absolute effect (95% CI)	Relative Risk or Mean Difference (95% CI)	Certainty of the evidence (GRADE)
Outcomes in infancy				
Survival without neurodisability at or beyond 12 months’ corrected age	4 studies^27–30^ (1019; HP:360, LP:659)	38 fewer per 1000 (from 76 fewer to 8 more)	RR 0.95 (0.90, 1.01)	⨁⨁◯◯ Low^a^
Survival to discharge or 36–40 weeks	11 studies^27,28,31–39^ (1675; HP:808, LP:867)	17 more per 1000 (from 9 fewer to 43 more)	RR 1.02 (0.99, 1.05)	⨁⨁⨁◯ Moderate^b^
Length in infancy	4 studies^34,55–57^ (728; HP: 229, LP: 499)	MD 0 cm (0.71 cm lower to 0.72 cm higher)		⨁⨁◯◯ Low^c^
Fat-free mass in infancy	1 study^51^ (58; HP: 37, LP: 21)	MD 16.7 g lower (253.96 g lower to 220.56 g higher)		⨁◯◯◯ Very low^d,e,f^
Outcomes during the toddler period				
Cognitive impairment or delay during the toddler period	2 studies^27,28^ (436; HP:215, LP:221)	50 more per 1000 (from 15 fewer to 153 more)	RR 1.36 (0.89, 2.09)	⨁⨁◯◯ Low^d,g^
Motor impairment or delay during the toddler period	2 studies^27,28^ (436; HP:215, LP:221)	10 more per 1000 (from 50 fewer to 99 more)	RR 1.06 (0.69, 1.61)	⨁⨁◯◯ Low^d,g^
Cerebral palsy during the toddler period	7 studies^10,27,28,31,41–43^ (1681; HP: 510, LP:1171)	19 more per 1000 (from 11 fewer to 70 more)	RR 1.35 (0.79, 2.30)	⨁⨁⨁◯ Moderate^h^
Length/height during the toddler period	6 studies^10,28,29,34,55^ (1,593; HP: 535, LP: 1058)	MD 0.13 cm lower (0.25 cm lower to 0.07 cm higher)		⨁⨁⨁◯ Moderate^i^
Outcomes during childhood				
Cerebral palsy during childhood	1 study^44^ (135; HP:67, LP:68)	102 fewer per 1000 (From 115 fewer to 1 fewer)	RR 0.13 (0.02, 0.99)	⨁⨁◯◯ Low^j,k^
Height in childhood	2 studies^55^(779; HP: 129, LP: 650)	MD 0.09 cm lower (1.62 cm lower to 0.35 cm higher)		⨁⨁◯◯ Low^l,m^

*CI* confidence interval, *RR* relative risk, *MD* mean difference, *HP* high protein, *LP* low protein.

^a^Downgraded two levels—some concerns/risk of bias for two studies and high risk of bias for one study.

^b^Downgraded one level—some concerns risk of bias for the included study (due to deviations from intended intervention, and selection of the reported results.

^c^Downgraded two levels—some concerns overall risk of bias for three studies and high overall risk of bias for one study.

^d^Downgraded one level—sample size too small to detect the differences.

^e^Downgraded at one level—some concerns overall risk of bias for one study.

^f^Downgraded at one level—only one study reported this outcome.

^g^Downgraded one level—some concerns overall risk of bias for one study due to randomisation, missing data for one of the two included studies.

^h^Downgraded one level—low event rate with small sample size

^i^Downgraded one level—some concerns overall risk of bias for four studies.

^j^Downgraded one level—some concerns risk of bias for the included study due to deviations from intended intervention, and selection of the reported results.

^k^Downgraded one level—small sample size and wide confidence interval.

^l^Downgraded one level—some concerns overall risk of bias for two studies.

^m^Downgraded one level—two included studies were conducted by the same team.

## Discussion

We conducted a systematic review and meta-analysis to assess the effects of planned high (≥3.5 g/kg/d) versus low protein intake after birth on later outcomes in children born preterm. In 44 studies that included data from 5338 children, we found no evidence of overall benefit of high protein intake for growth after the neonatal period and possibly harmful effects on survival, neurodisability and biochemical abnormalities in infancy. All the effect estimates in sensitivity analyses were similar to those of the overall analyses. However, there were few data beyond the toddler period, and findings were limited by moderate to low or very low-quality evidence, much of which did not meet traditional thresholds for statistical significance, from studies with considerable heterogeneity and bias.

Children born preterm have a higher risk of neurodevelopmental disabilities than term-born children,^77–79^ and it has been hypothesised that higher protein intake might be beneficial.^15^ However, we found little evidence of benefit. Rather, planned high protein intake may have increased the risk of neurodisability and cognitive impairment during infancy and the toddler period. The reason for these possible adverse effects is not clear, but in some cases insufficient energy intake may have contributed. At least two of the studies we reviewed delivered suboptimal energy to the study participants,^10,27^ and several studies did not specify the energy provided with the protein intervention. Optimal utilisation of amino acids depends on adequate energy intake.^80^ as excess amino acids are otherwise oxidised, causing oxidative stress by forming carbonyl groups (aldehydes and ketones).^81^ Studies have reported a positive association between oxidative stress and poor neurodevelopmental outcomes in preeclamptic mother-newborn dyads.^82^ and children born preterm.^83^ Thus, high protein intake may be deleterious where energy intake and capacity to metabolise the administered protein are insufficient.

Despite the finding of possible increased risk of neurodisability and cognitive impairment in infancy in HP group, there was little to no difference in the cognitive, language and motor scores during infancy and the toddler period between the HP and LP groups. One study also reported a reduced risk of neurodisability and cerebral palsy in childhood. There are several possible reasons for these apparently contradictory results. Firstly, the definition of neurodisability or neurological impairment varied across studies. For example, it was defined as any neurodisability in Bloomfield et al.^28^, severe mental retardation in Burattini et al.^29^, neurological impairment in Lucas et al.^30^ and composite scores <85 on all three Bayley scales in Balakrishnan et al.^27^ Secondly, studies reporting cognitive and language scores were highly heterogeneous, in part due to the use of different assessment tools and different versions of the same tool. Thirdly, for some of the outcomes the sample size was low and only two studies reported both the scores and numbers of children with impairments. Finally, assessment of cognitive skills and neurodisability in infancy is poorly predictive of later performance.^84,85^

Growth velocity in term-born healthy infants starts decelerating four to 6 months after birth, reaching a nadir in infancy, followed by a subtle slowing through mid-childhood.^86^ Children born preterm, at term-equivalent age, often have lower weight, length and head circumference but higher adiposity than their term-born counterparts.^87–90^ Many continue to show accelerated growth after initial postnatal growth faltering, maintaining this growth velocity until 36–40 weeks postmenstrual age, followed by a decrease and then a second increase in velocity during the infancy and toddler period.^86,90–93^ Consistent with this pattern, we found that children born preterm who received high protein intake had higher weight, fat mass and fat-free mass at discharge or 36 weeks and gained greater head circumference from birth to discharge or 36 weeks than those in the low protein group. However, this apparent growth benefit did not persist and children in the high protein intake group had smaller head circumferences and similar weight and length as those in the low protein group during infancy, toddler period and childhood. This suggests that high protein intake may have interfered with the expected second phase of catch-up growth, although the mechanisms underlying this effect are not clear.

In children born preterm, refeeding syndrome and related electrolyte disturbances are precipitated by the sudden supply of intravenous amino acids and glucose following a period of low nutrition.^11^ We found that infants in the high protein group had a higher risk of developing refeeding syndrome and its biochemical components, hypophosphatemia and hypercalcemia. Refeeding syndrome in neonates is associated with mortality and morbidity, including metabolic acidosis, hypernatremia, hypovolemia, ischaemia, respiratory alkalosis, sepsis and chronic lung disease.^11^ This suggests possible reasons why high protein intake contributed to the worse developmental outcomes we found in this study. Most of the studies reviewed were conducted in an era when electrolytes such as phosphate were withheld or restricted for the first few days after birth, likely exacerbating the effects of a higher protein intake to precipitate hypophosphatemia, though it is unknown if appropriate monitoring and treatment of electrolyte disturbances to prevent and treat refeeding syndrome may ameliorate these effects.

We found that children in the high protein group might have slightly higher serum albumin concentrations (mean difference 0.25 g/dl) than those in the LP group. Exogenous administration of amino acids stimulates organ-specific protein synthesis, and the introduction of amino acids immediately after birth stimulates albumin synthesis in preterm infants.^94^ Plasma albumin can temporarily store amino acids, preventing excess amino acid oxidation.^95^ We also found that HP intake group had a higher risk of having high blood urea concentration (>5 mmol/L) than their counterparts, likely reflecting oxidation of at least some of the additional amino acids.^96^ However, urea is considered as non-toxic at lower concentrations and, although the concentration at which serum urea can cause harm is yet to be determined, it has been recommended that lowering of protein intake should be considered if urea concentrations are above 5.7 mmol/L.^19^ Overall, these small biochemical changes related to the metabolism of protein are very unlikely to be of any clinical significance.

Children in the high protein intake group had a lower risk of hyperglycaemia than those in the low protein group. This may be because amino acids stimulate endogenous insulin secretion, potentially resulting in lower fasting blood glucose concentrations.^97,98^ Hyperglycaemia is associated with poor neurodevelopmental outcomes,^99^ and is difficult to manage in very preterm infants, either by decreasing glucose intake which risks deceleration of growth or treating with insulin with the associated risk of hypoglycemia.^99^ Hence, the lower risk of hyperglycaemia in infants with high protein intake might be of some clinical benefit for children born preterm.

Though we were able to extract data from large numbers of studies and children born preterm, we also found substantial unexplained heterogeneity. The heterogeneity might have resulted from variations in the effects of high protein intake in different sub-groups, variations in tools used and timing and assessment techniques of the reported outcomes. Moreover, the amino acid compositions of the provided protein interventions were highly variable as the interventions included fortified human milk, fortified bovine milk, preterm formula and amino acid solutions of varying compositions. The protein content of breast milk was also variably reported and in many studies was estimated rather than measured. In addition, the composition of mother’s own milk varies widely.^100^ Hence, the actual intake of protein from breast milk likely varied substantially within and between studies. Similarly, energy intake likely varied between studies and may have contributed to the unexplained heterogeneity in outcomes. In subgroup analyses, the effect of high protein intake appeared to vary only with route of intervention and time of study start and even then only for one outcome reported only by two studies, suggesting that these factors did not explain the degree of heterogeneity. However, there was limited power to detect the interactions due to insufficient data for many outcomes. Similarly, sensitivity analyses suggested that the heterogeneity was not explained by actual, as opposed to planned high or low protein intake, or by inclusion of studies at higher risk of bias. Although the effects of high protein intake on long-term outcomes may be expected to vary with gestational age and birth weight, most studies did not report outcomes in a way that allowed analysis of gestational age and birthweight subgroups. Individual participant data meta-analysis would be required further to explore sources of heterogeneity and effects in different subgroups.

The strengths of our study include lack of language or geographical restrictions and pooling of data from more than 5000 children. Limitations include insufficient data to conduct all pre-planned sub-group analyses or assess outcomes after the toddler period, low certainty of evidence for many outcomes that did not meet traditional thresholds for statistical significance and presence of unexplained heterogeneity.

## Conclusion

Planned high protein intake in the first weeks after preterm birth had few benefits and may be harmful for survival, neurodisability and biochemical abnormalities in neonatal care. However, there are few data beyond the toddler period and considerable unexplained heterogeneity. Longer-term follow-up and an individual participant data meta-analysis of existing trials, including data on total energy intake, would be helpful to clarify the effects of high protein intake for children born preterm.

## Supplementary information

SupplementaryFigures

Supplementary file 1

Supplementary table 1

Supplementary table 2

Supplementary table 3

Supplementary table 4

## Data Availability

Data access requests are to be submitted to the Data Access Committee via researchhub@auckland.ac.nz. Data will be shared with researchers with a sound proposal on reasonable request. The Liggins Institute reserves the right to charge a fee to cover the costs of making data available, if needed, for data requests that require additional work to prepare.
