# Correction: High protein intake on later outcomes in preterm children: a systematic review and meta-analysis

**DOI:** 10.1038/s41390-024-03592-8

**Published:** 2024-11-11

**Authors:** Subhasish Das, Thomas McClintock, Barbara E. Cormack, Frank H. Bloomfield, Jane E. Harding, Luling Lin

**Affiliations:** 1 Liggins Institute, University of Auckland, Auckland, New Zealand; 2 Nutrition Research Division, International Centre for Diarrhoeal Diseases Research, Bangladesh, Dhaka, Bangladesh; 3 Newborn Services, Starship Child Health, Auckland, New Zealand

Correction to: *Pediatric Research* 10.1038/s41390-024-03296-z, published online 10 June 2024

Some data errors were identified in the Supplementary Figs. 7b and 7c, as well as as in the “Results” section of the original article. The errors in the supplementary figures are detailed below.

Incorrect Supplementary Figs. 7b and 7c:
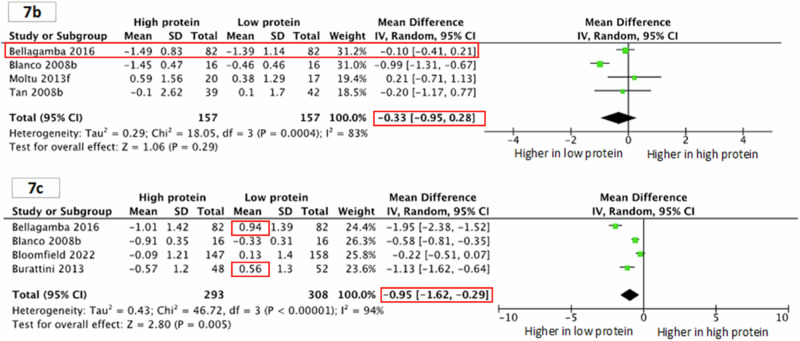


Corrected Supplementary Figs. 7b and 7c:
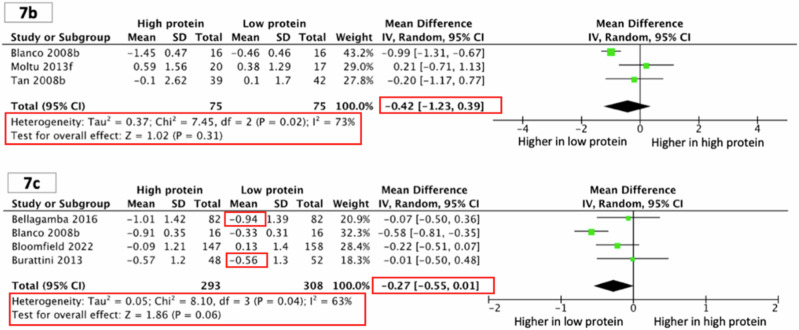


In the “Results” section (Growth: Head circumference) of the article, some data was also inadvertently incorrect. The following sentences

“However, HP group might have smaller head circumference z-scores during infancy (four studies.^10,45,57,58^; 314 children; MD -0.33, 95% CI −0.95, 0.28; P = 0.29; I^2^ 83%; Supplementary Fig. 7b) and the toddler period (four studies.^10,28,29,45^; 601 children; MD −0.95, 95% CI −1.62, −0.29; P = 0.005; I^2^ 94%; Supplementary Fig. 7c).”

have been corrected to:

“However, HP group might have smaller head circumference z-scores during infancy (three studies^45,57,58^; 150 children; MD −0.42, 95% CI −1.23, 0.39; P = 0.31; I^2^ 73%; Supplementary Fig. 7b) and the toddler period (four studies^10,28,29,45^; 601 children; MD −0.27, 95% CI −0.55, 0.01; P = 0.06; I^2^ 63%; Supplementary Fig. 7c)”.

The original article has been corrected.

## Supplementary information

Incorrect & Corrected Supplementary Figures 7b and 7c

