# Supplementary material for: High protein intake on later outcomes in preterm children: a systematic review and meta-analysis
Source: Pediatr Res. 2024 Jun 10;97(1):67–80. doi: 10.1038/s41390-024-03296-z (PMC11798874; doi:10.1038/s41390-024-03296-z)

Supplementary Fig. 1: Forest plots of effects of planned high vs. low protein intake on a) cognitive scores in infancy, b) cognitive scores during the toddler period, c) motor scores in infancy, and d) motor scores during the toddler period.

1a

Study or Subgroup	High protein Mean	SD	Total	Low protein Mean	SD	Total	Weight	Mean Difference IV, Random, 95% CI
Blanco 2008b	81	9	16	84	14	16	100.0%	−3.00 [−11.16, 5.16]
**Total (95% CI)**			**16**			**16**	**100.0%**	**−3.00 [−11.16, 5.16]**

Heterogeneity: Not applicable
Test for overall effect: Z = 0.72 (P = 0.47)

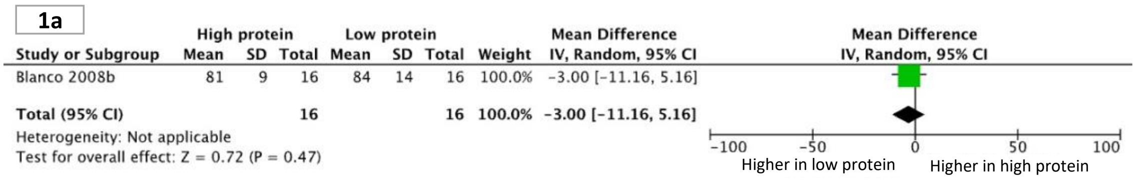

1b

Study or Subgroup	High protein Mean	SD	Total	Low protein Mean	SD	Total	Weight	Mean Difference IV, Random, 95% CI
Balakrishnan 2018	90.6	12.7	55	90.2	10.3	59	12.9%	0.40 [−3.86, 4.66]
Bellagamba 2016	94	13.9	76	93.8	12.9	73	12.8%	0.20 [−4.10, 4.50]
Blanco 2008a	57	11	16	63	13	16	5.8%	−6.00 [−14.34, 2.34]
Bloomfield 2022	94.2	15.7	159	95.7	14.4	162	15.5%	−1.50 [−4.80, 1.80]
Lucas 1989f	95.3	19.5	52	101.49	19.5	335	9.6%	−6.19 [−11.89, −0.49]
Lucas 1990a	98.6	18.4	59	101.37	16.86	251	10.8%	−2.77 [−7.91, 2.37]
Lucas 1996	106	2	125	103.8	2	120	22.3%	2.20 [1.70, 2.70]
Roelants 2018	97.14	13.45	34	97.85	12.27	69	10.3%	−0.71 [−6.08, 4.66]
**Total (95% CI)**			**576**			**1085**	**100.0%**	**−0.98 [−3.32, 1.35]**

Heterogeneity: Tau² = 6.32; Chi² = 21.80, df = 7 (P = 0.003); I² = 68%
Test for overall effect: Z = 0.82 (P = 0.41)

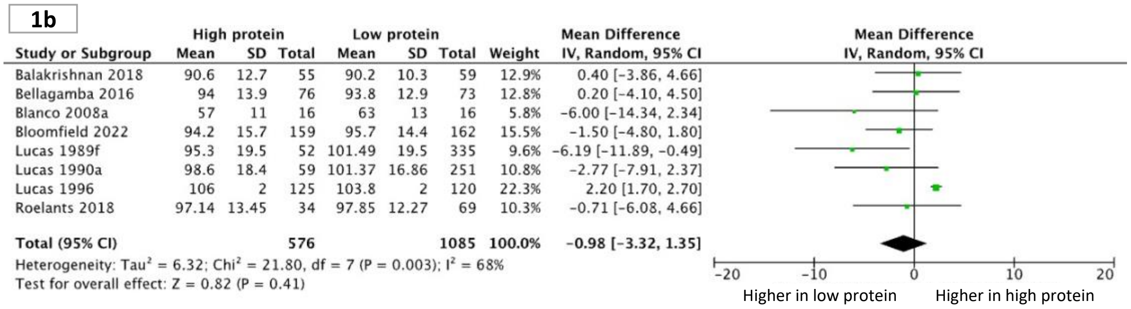

1c

Study or Subgroup	High protein Mean	SD	Total	Low protein Mean	SD	Total	Weight	Mean Difference IV, Random, 95% CI
Blanco 2008b	71	14	16	76	12	16	100.0%	−5.00 [−14.03, 4.03]
**Total (95% CI)**			**16**			**16**	**100.0%**	**−5.00 [−14.03, 4.03]**

Heterogeneity: Not applicable
Test for overall effect: Z = 1.08 (P = 0.28)

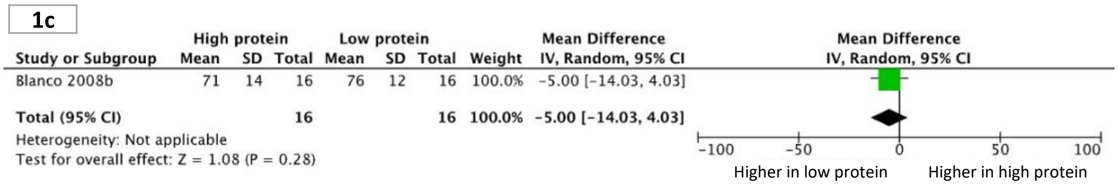

1d

Study or Subgroup	High protein Mean	SD	Total	Low protein Mean	SD	Total	Weight	Mean Difference IV, Random, 95% CI
Balakrishnan 2018	93	13.7	55	93.1	12.3	59	10.0%	−0.10 [−4.89, 4.69]
Bellagamba 2016	101	12.3	75	101.8	9.2	73	14.4%	−0.80 [−4.29, 2.69]
Blanco 2008a	67	15	16	64	12	16	3.5%	3.00 [−6.41, 12.41]
Bloomfield 2022	94.7	15.7	159	95.9	13	162	15.8%	−1.20 [−4.36, 1.96]
Lucas 1989f	94.2	15.9	52	94.62	15.48	335	10.4%	−0.42 [−5.05, 4.21]
Lucas 1990a	98.9	16.9	59	91.25	16.96	251	10.0%	7.65 [2.85, 12.45]
Lucas 1996	92.3	1.6	125	89.9	1.5	120	28.3%	2.40 [2.01, 2.79]
Roelants 2018	99.78	13.43	27	99.36	11.47	63	7.7%	0.42 [−5.38, 6.22]
**Total (95% CI)**			**568**			**1079**	**100.0%**	**1.22 [−0.66, 3.10]**

Heterogeneity: Tau² = 3.20; Chi² = 15.50, df = 7 (P = 0.03); I² = 55%
Test for overall effect: Z = 1.28 (P = 0.20)

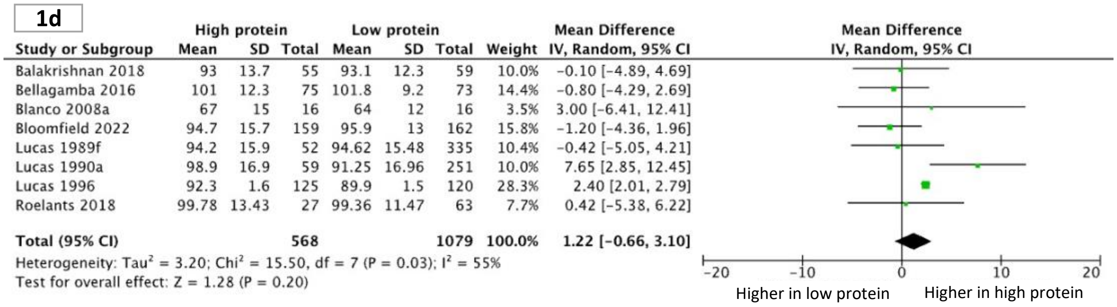

Supplementary Fig. 2: Forest plots of effects of planned high vs. low protein intake on weight a) at discharge or at 36 weeks (g), b) during infancy (kg), c) during the toddler period (kg), and d) during childhood (kg).

2a

Study or Subgroup	High protein Mean	SD	Total	Low protein Mean	SD	Total	Weight	Mean Difference IV, Random, 95% CI
Babson 1969	1,990	440	15	1,890	290	14	3.9%	100.00 [-169.55, 369.55]
Balakrishnan 2018	2,780	866	76	3,103	1,128	72	3.0%	-323.00 [-648.26, 2.26]
Balasubramanian 2013	1,371.58	202.64	60	1,494.7	224.4	63	10.2%	-123.12 [-198.61, -47.63]
Bellagamba 2016	1,936	299	82	1,958	345	82	9.3%	-22.00 [-120.81, 76.81]
Bloomfield 2022	2,440	423	180	2,372	413	182	9.8%	68.00 [-18.13, 154.13]
Brumberg 2010	2,279	567	11	1,995	572	12	1.7%	284.00 [-181.84, 749.84]
Burattini 2013	1,865	387	56	1,847	322	58	8.0%	18.00 [-112.92, 148.92]
Costa-Orvay 2011	2,048.75	192.66	32	1,903	223	6	5.9%	145.75 [-44.76, 336.26]
Ditzenberger 2013	2,279.23	421.12	30	2,336.13	414.06	34	5.5%	-56.90 [-262.03, 148.23]
Embleton 2005	2,548.45	369.22	51	2,263	468	26	5.4%	285.45 [78.98, 491.92]
Moltu 2013b	2,375	503	23	2,283	254	21	4.8%	92.00 [-140.51, 324.51]
Salas 2022	2,698	598	21	2,387	527	25	3.0%	311.00 [-17.77, 639.77]
Scattolin 2013	1,958.41	269.25	60	1,786.64	292.6	55	9.1%	171.77 [68.71, 274.83]
Tan 2008a	2,136	345	55	2,090	293	59	8.6%	46.00 [-71.91, 163.91]
Uthaya 2016	3,046.3	309.8	71	2,998.58	409.23	62	8.3%	47.72 [-77.06, 172.50]
Wilson 1997	2,111	904	64	2,080	809	61	3.4%	31.00 [-269.45, 331.45]
**Total (95% CI)**			**887**			**832**	**100.0%**	**51.80 [-13.76, 117.37]**

Heterogeneity: $Tau^2 = 9465.13$; $Chi^2 = 42.03$, df = 15 (P = 0.0002); $I^2 = 64\%$
Test for overall effect: Z = 1.55 (P = 0.12)

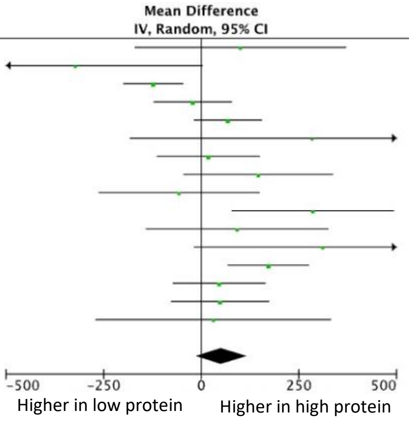

2b

Study or Subgroup	High protein Mean	SD	Total	Low protein Mean	SD	Total	Weight	Mean Difference IV, Random, 95% CI
Lucas 1989d	7.9	1.3	48	7.9	1.12	321	26.5%	0.00 [-0.39, 0.39]
Lucas 1996	8.09	1.12	125	8.01	1	123	57.1%	0.08 [-0.18, 0.34]
Moltu 2013d	6.99	0.88	17	6.98	1.115	13	7.3%	0.01 [-0.73, 0.75]
Tan 2008b	5.6	1.54	39	5.6	1.5	42	9.1%	0.00 [-0.66, 0.66]
**Total (95% CI)**			**229**			**499**	**100.0%**	**0.05 [-0.15, 0.25]**

Heterogeneity: $Tau^2 = 0.00$; $Chi^2 = 0.15$, df = 3 (P = 0.99); $I^2 = 0\%$
Test for overall effect: Z = 0.46 (P = 0.65)

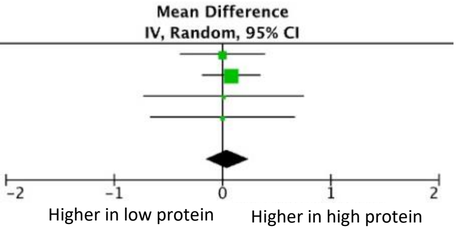

2c

Study or Subgroup	High protein Mean	SD	Total	Low protein Mean	SD	Total	Weight	Mean Difference IV, Random, 95% CI
Bellagamba 2016	11.61	1.4	82	11.72	1.8	82	10.6%	-0.11 [-0.60, 0.38]
Bloomfield 2022	11.85	1.5	159	12.22	1.7	162	21.0%	-0.37 [-0.72, -0.02]
Burattini 2013	11.69	1.9	48	11.82	1.7	52	5.1%	-0.13 [-0.84, 0.58]
Lucas 1989d	10	1.3	64	10.02	1.34	374	21.5%	-0.02 [-0.37, 0.33]
Lucas 1990e	10.4	1.6	61	10.28	1.2	273	14.2%	0.12 [-0.31, 0.55]
Lucas 1996	10.05	1.34	125	10.09	1.1	120	27.5%	-0.04 [-0.35, 0.27]
**Total (95% CI)**			**539**			**1063**	**100.0%**	**-0.09 [-0.25, 0.07]**

Heterogeneity: $Tau^2 = 0.00$; $Chi^2 = 3.66$, df = 5 (P = 0.60); $I^2 = 0\%$
Test for overall effect: Z = 1.15 (P = 0.25)

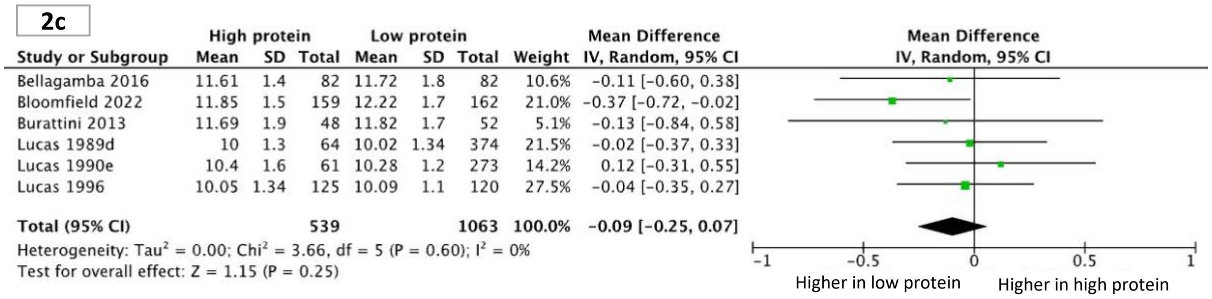

2d

Study or Subgroup	High protein Mean	SD	Total	Low protein Mean	SD	Total	Weight	Mean Difference IV, Random, 95% CI
Lucas 1989d	22.3	5.1	62	22.55	4.4	358	34.2%	-0.25 [-1.60, 1.10]
Lucas 1990e	23.3	3.5	67	23.51	4.29	292	65.8%	-0.21 [-1.18, 0.76]
**Total (95% CI)**			**129**			**650**	**100.0%**	**-0.22 [-1.01, 0.56]**

Heterogeneity: $Tau^2 = 0.00$; $Chi^2 = 0.00$, df = 1 (P = 0.96); $I^2 = 0\%$
Test for overall effect: Z = 0.56 (P = 0.58)

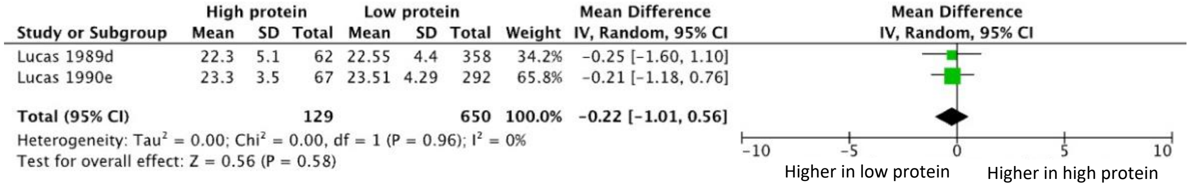

Supplementary Fig. 3: Forest plots of effects of planned high vs. low protein intake on weight Z-score a) at discharge or at 36 weeks, b) during infancy, c) during the toddler period, and d) gain in weight z-score to discharge or to 36 weeks.

3a

Study or Subgroup	High protein Mean	SD	Total	Low protein Mean	SD	Total	Weight	Mean Difference IV, Random, 95% CI
Balakrishnan 2018	−1.07	0.82	72	−0.71	0.94	72	11.0%	−0.36 [−0.65, −0.07]
Bellagamba 2016	−1.73	0.73	82	−1.68	0.85	82	12.3%	−0.05 [−0.29, 0.19]
Bloomfield 2022	−0.65	0.95	180	−0.68	0.98	182	13.7%	0.03 [−0.17, 0.23]
Burattini 2013	−1.88	0.93	56	−1.95	0.8	58	10.1%	0.07 [−0.25, 0.39]
Costa-Orvay 2011	−1.45	0.79	32	−1.567	0.74	6	4.3%	0.12 [−0.54, 0.77]
Embleton 2005	−0.8	0.92	51	−1.3	0.9	26	7.5%	0.50 [0.07, 0.93]
Moltu 2013b	−1.24	1.07	23	−1.46	0.5	21	6.4%	0.22 [−0.27, 0.71]
Morgan 2014a	−1.41	0.72	62	−1.68	0.8	62	11.5%	0.27 [0.00, 0.54]
Roelants 2018	−1.27	0.91	45	−1.6	1.1	89	9.3%	0.33 [−0.02, 0.68]
Salas 2022	0	1.2	21	−0.9	1.3	25	3.7%	0.90 [0.18, 1.62]
Tan 2008a	−1.3	0.9	55	−1.4	0.8	59	10.2%	0.10 [−0.21, 0.41]
**Total (95% CI)**			**679**			**682**	**100.0%**	**0.13 [−0.03, 0.28]**

Heterogeneity: $Tau^2 = 0.04$; $Chi^2 = 23.16$, df = 10 (P = 0.01); $I^2 = 57\%$
Test for overall effect: Z = 1.61 (P = 0.11)

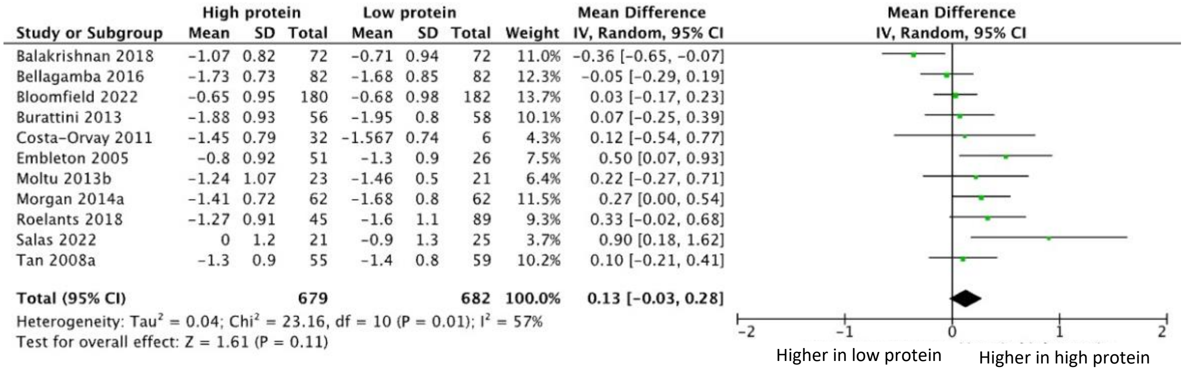

3b

Study or Subgroup	High protein Mean	SD	Total	Low protein Mean	SD	Total	Weight	Mean Difference IV, Random, 95% CI
Blanco 2008b	−1.62	0.46	16	−0.76	0.43	16	40.6%	−0.86 [−1.17, −0.55]
Moltu 2013f	−0.77	1.08	20	−0.76	0.43	17	34.5%	−0.01 [−0.53, 0.51]
Tan 2008b	−0.6	1.69	39	−0.4	2.14	42	24.9%	−0.20 [−1.04, 0.64]
**Total (95% CI)**			**75**			**75**	**100.0%**	**−0.40 [−1.03, 0.22]**

Heterogeneity: $Tau^2 = 0.23$; $Chi^2 = 8.64$, df = 2 (P = 0.01); $I^2 = 77\%$
Test for overall effect: Z = 1.26 (P = 0.21)

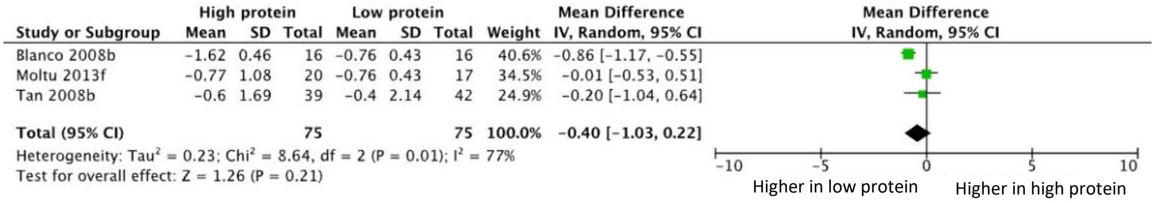

3c

Study or Subgroup	High protein Mean	SD	Total	Low protein Mean	SD	Total	Weight	Mean Difference IV, Random, 95% CI
Bellagamba 2016	−0.09	0.96	82	−0.1	1.31	82	17.8%	0.01 [−0.34, 0.36]
Blanco 2008b	−0.82	0.41	16	−0.22	0.35	16	19.7%	−0.60 [−0.86, −0.34]
Bloomfield 2022	−0.24	1.09	159	−0.05	1.14	162	20.1%	−0.19 [−0.43, 0.05]
Burattini 2013	−0.22	1.31	48	−0.17	1.12	52	14.9%	−0.05 [−0.53, 0.43]
Moltu 2013b	−0.85	1.11	20	−0.66	1.04	17	10.7%	−0.19 [−0.88, 0.50]
Roelants 2018	0.06	1.05	37	−0.42	0.86	74	16.9%	0.48 [0.09, 0.87]
**Total (95% CI)**			**362**			**403**	**100.0%**	**−0.10 [−0.41, 0.21]**

Heterogeneity: $Tau^2 = 0.11$; $Chi^2 = 22.07$, df = 5 (P = 0.0005); $I^2 = 77\%$
Test for overall effect: Z = 0.64 (P = 0.52)

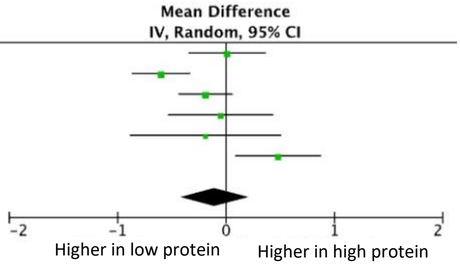

3d

Study or Subgroup	High protein Mean	SD	Total	Low protein Mean	SD	Total	Weight	Mean Difference IV, Random, 95% CI
Moltu 2013b	0.08	0.48	23	0.56	0.51	21	14.2%	−0.48 [−0.77, −0.19]
Bloomfield 2022	−0.6	0.78	180	−0.57	0.88	182	19.1%	−0.03 [−0.20, 0.14]
Shah 2016	−1.28	0.57	49	−1.41	0.47	50	17.7%	0.13 [−0.08, 0.34]
Embleton 2005	0.65	0.5	51	0.5	0.4	26	17.7%	0.15 [−0.06, 0.36]
Vlaardingerbroek 2013	−0.03	1.3	47	−0.2	1.3	97	9.3%	0.17 [−0.28, 0.62]
Salas 2022	−0.6	0.7	21	−0.8	0.8	25	9.7%	0.20 [−0.23, 0.63]
Roelants 2018	−1.27	0.91	45	−1.6	1.09	89	12.2%	0.33 [−0.02, 0.68]
**Total (95% CI)**			**416**			**490**	**100.0%**	**0.05 [−0.13, 0.23]**

Heterogeneity: $Tau^2 = 0.04$; $Chi^2 = 18.03$, df = 6 (P = 0.006); $I^2 = 67\%$
Test for overall effect: Z = 0.56 (P = 0.57)

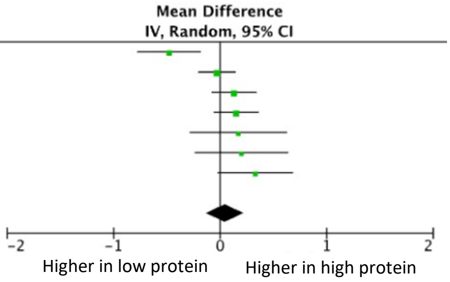

Supplementary Fig. 4: Forest plots of effects of planned high vs. low protein intake on length (in cm) a) at discharge or at 36 weeks, b) during infancy, c) during the toddler period and d) during childhood.

4a

	High protein			Low protein				Mean Difference
Study or Subgroup	Mean	SD	Total	Mean	SD	Total	Weight	IV, Random, 95% CI
Babson 1969	44.82	2.56	15	44.24	2.11	14	3.4%	0.58 [−1.12, 2.28]
Balakrishnan 2018	46.5	4.1	74	48	4.2	70	4.7%	−1.50 [−2.86, −0.14]
Balasubramanian 2013	39.19	1.8	60	40.21	2.34	63	8.6%	−1.02 [−1.76, −0.28]
Bellagamba 2016	42.7	2.3	82	42.8	2	82	9.2%	−0.10 [−0.76, 0.56]
Bloomfield 2022	43.8	3	177	43.4	2.6	173	9.9%	0.40 [−0.19, 0.99]
Burattini 2013	42.7	2.4	56	42.7	1.9	58	8.1%	0.00 [−0.80, 0.80]
Costa-Orvay 2011	44.99	1.45	32	44.2	1.52	6	4.9%	0.79 [−0.53, 2.11]
Embleton 2005	45.5	2.3	51	44	2	26	6.7%	1.50 [0.51, 2.49]
Kanmaz 2013	41.7	2.32	29	41.9	2.23	55	6.5%	−0.20 [−1.23, 0.83]
Kashyap 1988	44.35	0.92	30	44.2	0.81	14	10.3%	0.15 [−0.39, 0.69]
Moltu 2013b	43.9	3.15	21	43.3	1.96	20	3.8%	0.60 [−1.00, 2.20]
Scattolin 2013	43.06	2.19	60	42.03	2.19	55	8.1%	1.03 [0.23, 1.83]
Tan 2008a	42.9	2.3	55	42.4	2.1	59	8.0%	0.50 [−0.31, 1.31]
Uthaya 2016	48.67	3.26	71	47.83	3.8	62	5.4%	0.84 [−0.37, 2.05]
Wilson 1997	43	6.3	64	42.8	5.9	61	2.4%	0.20 [−1.94, 2.34]
**Total (95% CI)**			**877**			**818**	**100.0%**	**0.23 [−0.14, 0.60]**

Heterogeneity: Tau² = 0.26; Chi² = 32.17, df = 14 (P = 0.004); I² = 56%
Test for overall effect: Z = 1.23 (P = 0.22)

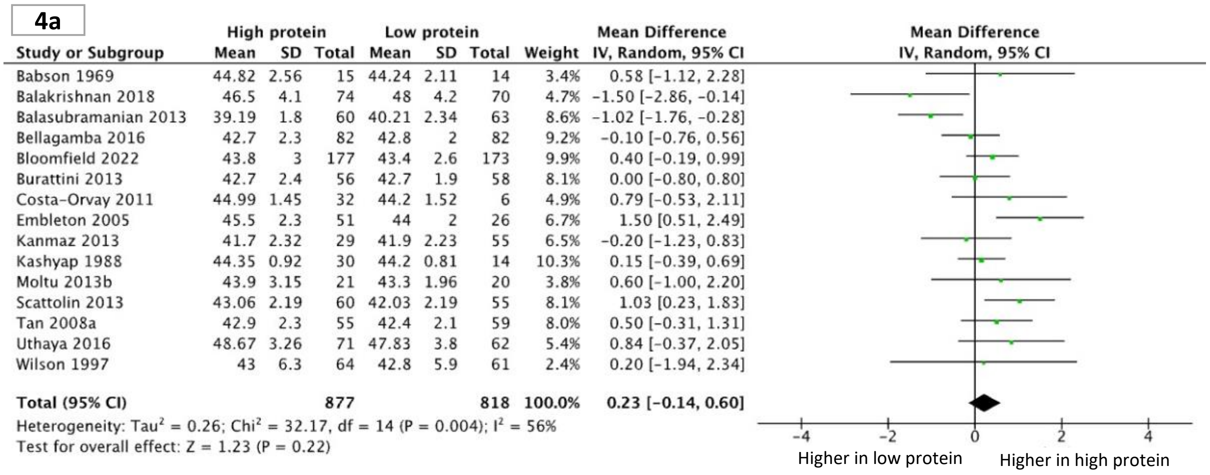

4b

	High protein			Low protein				Mean Difference
Study or Subgroup	Mean	SD	Total	Mean	SD	Total	Weight	IV, Random, 95% CI
Lucas 1989d	69.2	3.7	48	69.32	3.22	321	41.8%	−0.12 [−1.22, 0.98]
Lucas 1996	69.8	3.35	125	69.3	5.55	123	39.0%	0.50 [−0.64, 1.64]
Moltu 2013d	66.2	2.5	17	66.7	3.1	13	12.0%	−0.50 [−2.56, 1.56]
Tan 2008b	57.73	6.4	39	58.9	5.83	42	7.1%	−1.17 [−3.84, 1.50]
**Total (95% CI)**			**229**			**499**	**100.0%**	**0.00 [−0.71, 0.72]**

Heterogeneity: Tau² = 0.00; Chi² = 1.74, df = 3 (P = 0.63); I² = 0%
Test for overall effect: Z = 0.00 (P = 1.00)

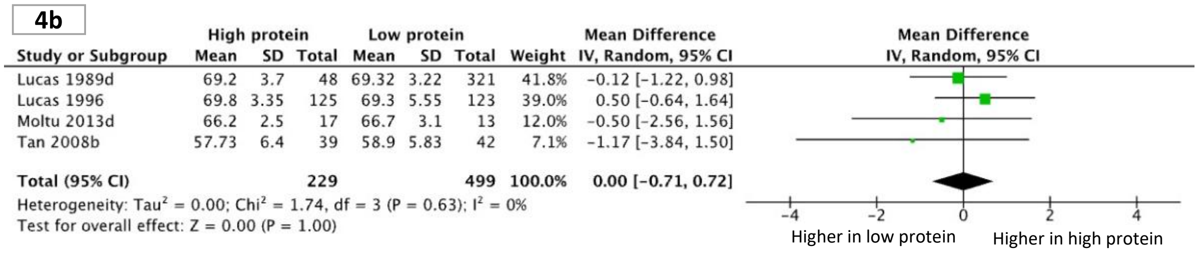

4c

	High protein			Low protein				Mean Difference
Study or Subgroup	Mean	SD	Total	Mean	SD	Total	Weight	IV, Random, 95% CI
Bellagamba 2016	86.8	3.4	82	87.4	4.4	82	13.6%	−0.60 [−1.80, 0.60]
Bloomfield 2022	85.9	3.5	155	86.7	4.2	157	22.8%	−0.80 [−1.66, 0.06]
Burattini 2013	87.3	4.6	48	87.4	4.1	52	7.4%	−0.10 [−1.81, 1.61]
Lucas 1989d	79.3	3.7	64	79.15	3.6	374	18.8%	0.15 [−0.83, 1.13]
Lucas 1990e	80.9	4.5	61	79.94	3.33	273	13.7%	0.96 [−0.24, 2.16]
Lucas 1996	80	3.35	125	80.1	3.29	120	23.7%	−0.10 [−0.93, 0.73]
**Total (95% CI)**			**535**			**1058**	**100.0%**	**−0.13 [−0.62, 0.35]**

Heterogeneity: Tau² = 0.08; Chi² = 6.43, df = 5 (P = 0.27); I² = 22%
Test for overall effect: Z = 0.54 (P = 0.59)

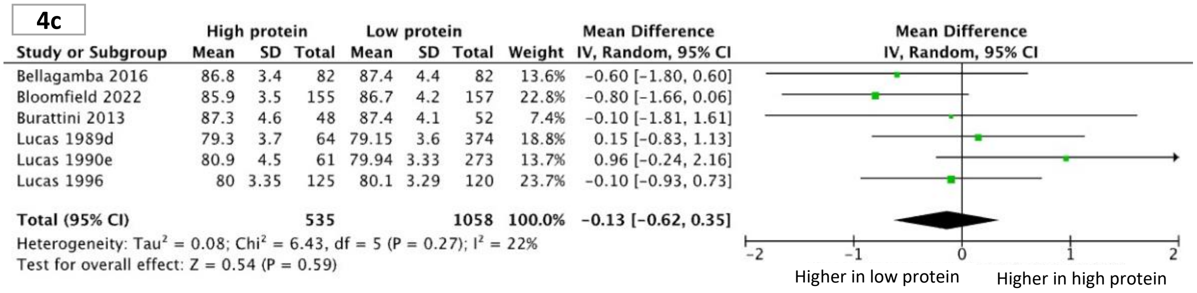

4d

	High protein			Low protein				Mean Difference
Study or Subgroup	Mean	SD	Total	Mean	SD	Total	Weight	IV, Random, 95% CI
Lucas 1989d	120.4	6.6	62	121.1	6.2	358	41.6%	−0.70 [−2.46, 1.06]
Lucas 1990e	122.9	5.5	67	122.56	6.04	292	58.4%	0.34 [−1.15, 1.83]
**Total (95% CI)**			**129**			**650**	**100.0%**	**−0.09 [−1.23, 1.04]**

Heterogeneity: Tau² = 0.00; Chi² = 0.78, df = 1 (P = 0.38); I² = 0%
Test for overall effect: Z = 0.16 (P = 0.87)

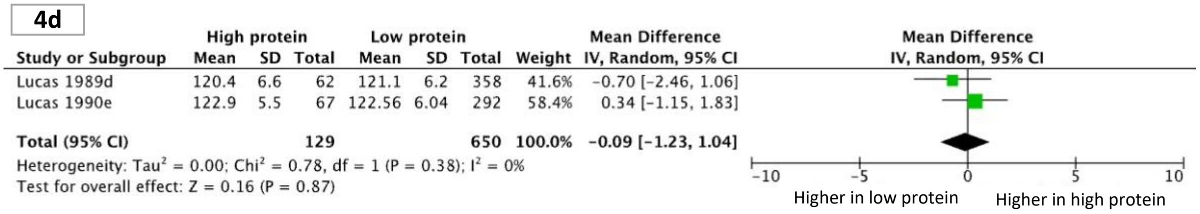

Supplementary Fig. 5: Forest plots of effects of planned high vs. low protein intake on length Z-score a) at discharge or at 36 weeks, b) during infancy, c) during the toddler period, and d) gain in length z-score to discharge or to 36 weeks.

5a

Study or Subgroup	High protein Mean	SD	Total	Low protein Mean	SD	Total	Weight	Mean Difference IV, Random, 95% CI
Balakrishnan 2018	−1.39	1.07	70	−1.04	1.28	70	11.9%	−0.35 [−0.74, 0.04]
Bellagamba 2016	−1.74	0.88	82	−1.7	0.8	82	18.1%	−0.04 [−0.30, 0.22]
Bloomfield 2022	−1.32	1.17	177	−1.34	1.02	173	19.7%	0.02 [−0.21, 0.25]
Burattini 2013	−1.82	0.9	56	−1.86	0.76	58	15.5%	0.04 [−0.27, 0.35]
Costa-Orvay 2011	−0.55	0.77	32	−0.89	0.84	6	4.8%	0.34 [−0.38, 1.06]
Embleton 2005	−1.45	0.99	51	−2	0.9	26	10.2%	0.55 [0.11, 0.99]
Moltu 2013b	−1.19	1.1	21	−1.48	0.89	20	6.3%	0.29 [−0.32, 0.90]
Salas 2022	−1.4	1.3	21	−2	1.6	25	3.7%	0.60 [−0.24, 1.44]
Tan 2008a	−2.3	1.3	55	−2.6	1.2	59	9.6%	0.30 [−0.16, 0.76]
**Total (95% CI)**			**565**			**519**	**100.0%**	**0.10 [−0.07, 0.28]**

Heterogeneity: $Tau^2 = 0.03$; $Chi^2 = 13.46$, df = 8 (P = 0.10); $I^2 = 41\%$
Test for overall effect: Z = 1.17 (P = 0.24)

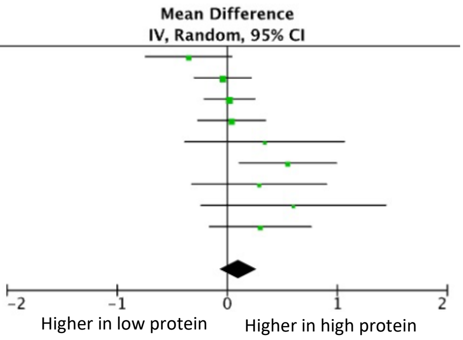

5b

Study or Subgroup	High protein Mean	SD	Total	Low protein Mean	SD	Total	Weight	Mean Difference IV, Random, 95% CI
Blanco 2008b	−1.44	0.47	16	−0.52	0.47	16	39.7%	−0.92 [−1.25, −0.59]
Moltu 2013f	−0.42	1.12	20	−0.77	0.94	17	34.5%	0.35 [−0.31, 1.01]
Tan 2008b	−0.9	2.62	39	−0.8	2.6	42	25.8%	−0.10 [−1.24, 1.04]
**Total (95% CI)**			**75**			**75**	**100.0%**	**−0.27 [−1.21, 0.67]**

Heterogeneity: $Tau^2 = 0.55$; $Chi^2 = 12.25$, df = 2 (P = 0.002); $I^2 = 84\%$
Test for overall effect: Z = 0.57 (P = 0.57)

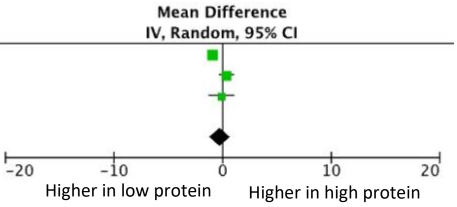

5c

Study or Subgroup	High protein Mean	SD	Total	Low protein Mean	SD	Total	Weight	Mean Difference IV, Random, 95% CI
Bellagamba 2016	0.66	1.02	82	0.79	1.34	82	20.6%	−0.13 [−0.49, 0.23]
Blanco 2008b	−0.85	0.38	16	−0.26	0.35	16	24.5%	−0.59 [−0.84, −0.34]
Bloomfield 2022	−0.55	1.07	155	−0.41	1.18	157	24.6%	−0.14 [−0.39, 0.11]
Burattini 2013	0.61	1.25	48	0.57	1.12	52	17.2%	0.04 [−0.43, 0.51]
Moltu 2013f	−0.46	0.92	20	−0.88	0.98	17	13.1%	0.42 [−0.20, 1.04]
**Total (95% CI)**			**321**			**324**	**100.0%**	**−0.14 [−0.44, 0.15]**

Heterogeneity: $Tau^2 = 0.08$; $Chi^2 = 14.10$, df = 4 (P = 0.007); $I^2 = 72\%$
Test for overall effect: Z = 0.94 (P = 0.34)

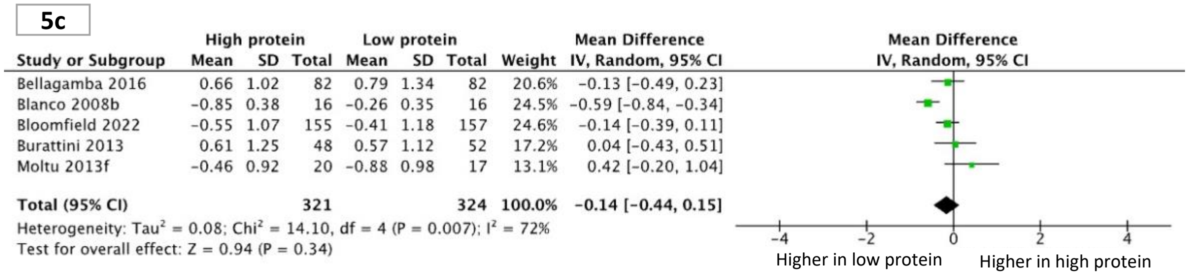

5d

Study or Subgroup	High protein Mean	SD	Total	Low protein Mean	SD	Total	Weight	Mean Difference IV, Random, 95% CI
Bloomfield 2022	−1.2	1.23	173	−1.15	1	167	38.3%	−0.05 [−0.29, 0.19]
Embleton 2005	0.6	0.5	51	0.5	0.5	26	38.8%	0.10 [−0.14, 0.34]
Moltu 2013b	−0.31	1.11	21	−0.82	0.87	19	5.9%	0.51 [−0.11, 1.13]
Shah 2016	−1.58	0.93	49	−1.59	0.89	50	17.1%	0.01 [−0.35, 0.37]
**Total (95% CI)**			**294**			**262**	**100.0%**	**0.05 [−0.10, 0.20]**

Heterogeneity: $Tau^2 = 0.00$; $Chi^2 = 3.05$, df = 3 (P = 0.38); $I^2 = 2\%$
Test for overall effect: Z = 0.67 (P = 0.50)

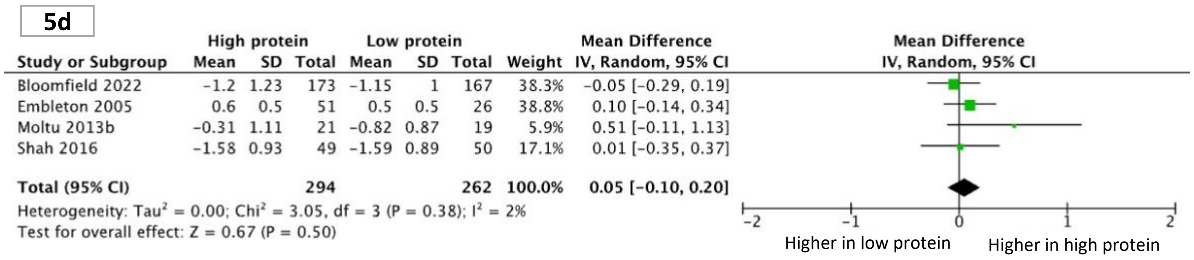

Supplementary Fig. 6: Forest plots of effects of planned high vs. low protein intake on head circumference (in cm) a) at discharge or at 36 weeks, b) during infancy, c) during toddler period, d) during childhood and e) gain in head circumference till discharge or 36 weeks.

6a

	High protein			Low protein				Mean Difference
Study or Subgroup	Mean	SD	Total	Mean	SD	Total	Weight	IV, Random, 95% CI
Balakrishnan 2018	33.2	2.8	75	34.2	3	70	4.9%	−1.00 [−1.95, −0.05]
Balasubramanian 2013	28	1.52	60	29	2.28	63	6.8%	−1.00 [−1.68, −0.32]
Bellagamba 2016	30.6	1.2	82	30.8	1.6	82	9.0%	−0.20 [−0.63, 0.23]
Bloomfield 2022	31.3	1.6	180	31.1	1.5	176	10.0%	0.20 [−0.12, 0.52]
Burattini 2013	30.5	1.4	56	30.6	1.3	58	8.4%	−0.10 [−0.60, 0.40]
Costa−Orvay 2011	32.25	0.88	32	31.5	1.28	6	4.2%	0.75 [−0.32, 1.82]
Ditzenberger 2013	31.8	1.56	30	32.4	2.2	34	5.0%	−0.60 [−1.53, 0.33]
Embleton 2005	33.8	1.6	51	32.9	1.7	26	5.9%	0.90 [0.11, 1.69]
Kashyap 1988	32.6	0.7	30	33.1	0.79	14	8.5%	−0.50 [−0.98, −0.02]
Moltu 2013b	33.4	1.5	22	32.5	1.41	21	5.4%	0.90 [0.03, 1.77]
Morgan 2014a	31.6	1.3	63	31.1	1.5	63	8.4%	0.50 [0.01, 0.99]
Scattolin 2013	30.85	1.34	60	30.71	1.94	55	7.3%	0.14 [−0.47, 0.75]
Tan 2008a	31.1	1.5	55	31.4	1.3	59	8.2%	−0.30 [−0.82, 0.22]
Uthaya 2016	35	1.9	71	35.7	2.7	62	5.8%	−0.70 [−1.50, 0.10]
Wilson 1997	31.4	5	64	31.5	4.5	61	2.2%	−0.10 [−1.77, 1.57]
**Total (95% CI)**			**931**			**850**	**100.0%**	**−0.08 [−0.35, 0.20]**

Heterogeneity: $Tau^2 = 0.17$; $Chi^2 = 39.76$, df = 14 (P = 0.0003); $I^2 = 65\%$
Test for overall effect: Z = 0.55 (P = 0.58)

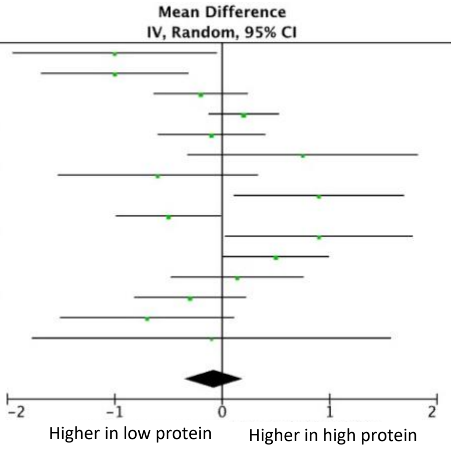

6b

	High protein			Low protein				Mean Difference
Study or Subgroup	Mean	SD	Total	Mean	SD	Total	Weight	IV, Random, 95% CI
Lucas 1989d	45.3	1.8	48	45.5	1.57	321	24.2%	−0.20 [−0.74, 0.34]
Lucas 1996	45.7	1.12	125	45.5	1.11	123	48.6%	0.20 [−0.08, 0.48]
Moltu 2013d	43.4	1.1	18	43.8	0.5	13	21.9%	−0.40 [−0.98, 0.18]
Tan 2008b	40.3	2.92	39	40.5	3.22	42	5.3%	−0.20 [−1.54, 1.14]
**Total (95% CI)**			**230**			**499**	**100.0%**	**−0.05 [−0.37, 0.27]**

Heterogeneity: $Tau^2 = 0.03$; $Chi^2 = 4.40$, df = 3 (P = 0.22); $I^2 = 32\%$
Test for overall effect: Z = 0.30 (P = 0.76)

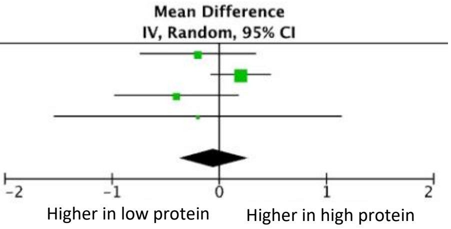

6c

	High protein			Low protein				Mean Difference
Study or Subgroup	Mean	SD	Total	Mean	SD	Total	Weight	IV, Random, 95% CI
Bellagamba 2016	47.6	1.94	82	47.9	1.77	82	13.6%	−0.30 [−0.87, 0.27]
Bloomfield 2022	47.7	1.7	147	48.1	2	158	25.5%	−0.40 [−0.82, 0.02]
Burattini 2013	48.1	1.9	48	48.4	1.6	52	9.2%	−0.30 [−0.99, 0.39]
Lucas 1989d	47.7	1.5	64	48.04	1.59	374	27.3%	−0.34 [−0.74, 0.06]
Lucas 1990e	47.7	1.5	61	47.97	1.66	273	24.4%	−0.27 [−0.69, 0.15]
**Total (95% CI)**			**402**			**939**	**100.0%**	**−0.33 [−0.54, −0.12]**

Heterogeneity: $Tau^2 = 0.00$; $Chi^2 = 0.21$, df = 4 (P = 1.00); $I^2 = 0\%$
Test for overall effect: Z = 3.07 (P = 0.002)

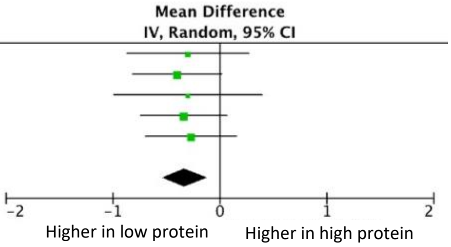

6d

	High protein			Low protein				Mean Difference
Study or Subgroup	Mean	SD	Total	Mean	SD	Total	Weight	IV, Random, 95% CI
Lucas 1989d	51.9	1.5	62	52.24	1.92	358	47.7%	−0.34 [−0.76, 0.08]
Lucas 1990e	52.1	1.5	67	52.48	1.61	292	52.3%	−0.38 [−0.78, 0.02]
**Total (95% CI)**			**129**			**650**	**100.0%**	**−0.36 [−0.65, −0.07]**

Heterogeneity: $Tau^2 = 0.00$; $Chi^2 = 0.02$, df = 1 (P = 0.89); $I^2 = 0\%$
Test for overall effect: Z = 2.42 (P = 0.02)

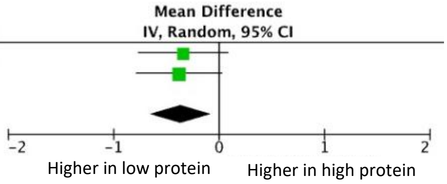

6e

	High protein			Low protein				Mean Difference
Study or Subgroup	Mean	SD	Total	Mean	SD	Total	Weight	IV, Random, 95% CI
Balasubramanian 2013	0.29	0.42	60	0.62	0.38	63	5.5%	−0.33 [−0.47, −0.19]
Brumberg 2010	1.13	0.31	11	0.82	0.36	12	2.0%	0.31 [0.04, 0.58]
Gupta 2020	0.97	0.19	75	0.9	0.2	73	11.3%	0.07 [0.01, 0.13]
Kanmaz 2013	0.82	0.21	29	0.81	0.24	55	8.1%	0.01 [−0.09, 0.11]
Kashyap 1986	1.2	0.25	18	0.85	0.15	9	5.0%	0.35 [0.20, 0.50]
Kashyap 1988	1.2	0.25	30	0.98	0.11	14	7.6%	0.22 [0.11, 0.33]
Kim 2015	1	0.05	66	1	0.04	63	15.0%	0.00 [−0.02, 0.02]
Lucas 1996	1.01	0.035	137	0.95	0.03	138	15.2%	0.06 [0.05, 0.07]
Moro 1991	0.96	0.25	9	1.02	0.36	12	2.2%	−0.06 [−0.32, 0.20]
Polberger 1989	1.15	0.14	14	1.01	0.22	14	5.7%	0.14 [0.00, 0.28]
Thanh 2022	0.94	0.25	70	0.92	0.26	75	9.5%	0.02 [−0.06, 0.10]
Vlaardingerbroek 2013	0.84	0.13	47	0.82	0.14	97	12.8%	0.02 [−0.03, 0.07]
**Total (95% CI)**			**566**			**625**	**100.0%**	**0.05 [0.01, 0.09]**

Heterogeneity: $Tau^2 = 0.00$; $Chi^2 = 107.12$, df = 11 (P < 0.00001); $I^2 = 90\%$
Test for overall effect: Z = 2.44 (P = 0.01)

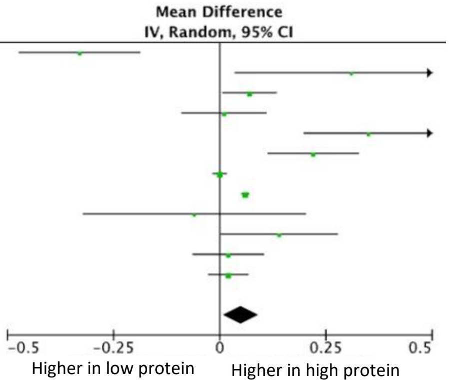

Supplementary Fig. 7: Forest plots of effects of planned high vs. low protein intake on head circumference z-score a) at discharge or at 36 weeks, b) during infancy, c) during the toddler period, d) gain in head circumference z-score to discharge or to 36 weeks.

7a

Study or Subgroup	High protein Mean	SD	Total	Low protein Mean	SD	Total	Weight	Mean Difference IV, Random, 95% CI
Balakrishnan 2018	−0.65	0.76	70	−0.34	0.91	70	14.9%	−0.31 [−0.59, −0.03]
Bellagamba 2016	−1.49	0.83	82	−1.39	1.14	82	13.7%	−0.10 [−0.41, 0.21]
Bloomfield 2022	−0.88	1	180	−0.93	1.01	176	18.2%	0.05 [−0.16, 0.26]
Burattini 2013	−1.59	0.88	56	−1.53	0.9	58	12.9%	−0.06 [−0.39, 0.27]
Costa-Orvay 2011	−0.51	0.66	32	−0.65	0.55	6	7.8%	0.14 [−0.36, 0.64]
Moltu 2013b	0.4	0.82	22	0.02	0.71	21	8.7%	0.38 [−0.08, 0.84]
Morgan 2014a	−0.93	1.06	63	−1.32	1.18	63	10.6%	0.39 [−0.00, 0.78]
Salas 2022	−1.2	1	21	−1.8	1.8	25	3.5%	0.60 [−0.23, 1.43]
Tan 2008a	−1	1.2	55	−0.8	1.1	59	9.6%	−0.20 [−0.62, 0.22]
**Total (95% CI)**			**581**			**560**	**100.0%**	**0.03 [−0.14, 0.20]**

Heterogeneity: $Tau^2 = 0.03$; $Chi^2 = 15.19$, df = 8 (P = 0.06); $I^2 = 47\%$
Test for overall effect: Z = 0.34 (P = 0.74)

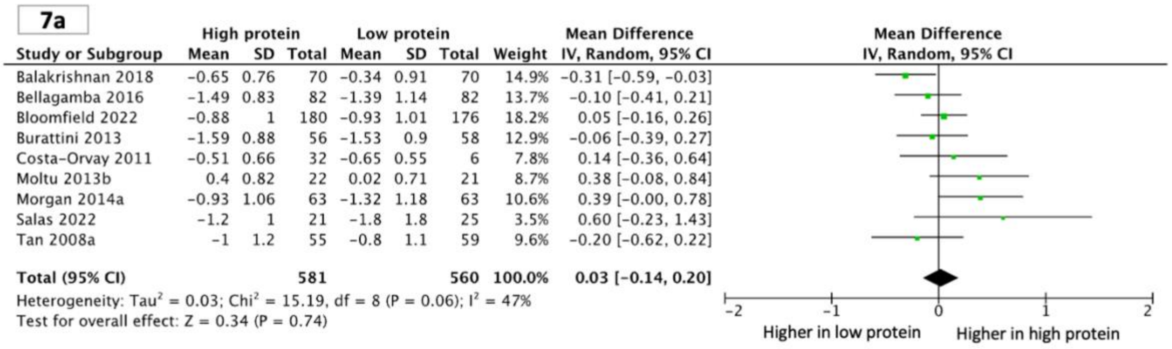

7b

Study or Subgroup	High protein Mean	SD	Total	Low protein Mean	SD	Total	Weight	Mean Difference IV, Random, 95% CI
Blanco 2008b	−1.45	0.47	16	−0.46	0.46	16	43.2%	−0.99 [−1.31, −0.67]
Moltu 2013f	0.59	1.56	20	0.38	1.29	17	29.0%	0.21 [−0.71, 1.13]
Tan 2008b	−0.1	2.62	39	0.1	1.7	42	27.8%	−0.20 [−1.17, 0.77]
**Total (95% CI)**			**75**			**75**	**100.0%**	**−0.42 [−1.23, 0.39]**

Heterogeneity: $Tau^2 = 0.37$; $Chi^2 = 7.45$, df = 2 (P = 0.02); $I^2 = 73\%$
Test for overall effect: Z = 1.02 (P = 0.31)

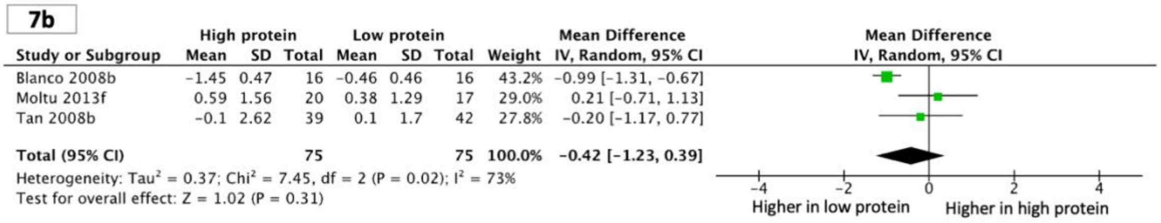

7c

Study or Subgroup	High protein Mean	SD	Total	Low protein Mean	SD	Total	Weight	Mean Difference IV, Random, 95% CI
Bellagamba 2016	−1.01	1.42	82	−0.94	1.39	82	20.9%	−0.07 [−0.50, 0.36]
Blanco 2008b	−0.91	0.35	16	−0.33	0.31	16	32.3%	−0.58 [−0.81, −0.35]
Bloomfield 2022	−0.09	1.21	147	0.13	1.4	158	28.4%	−0.22 [−0.51, 0.07]
Burattini 2013	−0.57	1.2	48	−0.56	1.3	52	18.3%	−0.01 [−0.50, 0.48]
**Total (95% CI)**			**293**			**308**	**100.0%**	**−0.27 [−0.55, 0.01]**

Heterogeneity: $Tau^2 = 0.05$; $Chi^2 = 8.10$, df = 3 (P = 0.04); $I^2 = 63\%$
Test for overall effect: Z = 1.86 (P = 0.06)

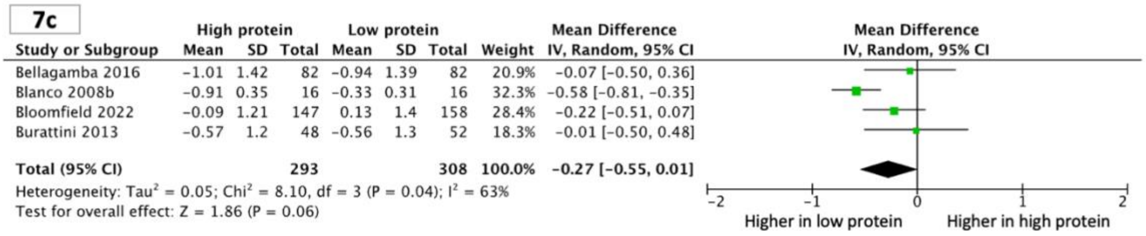

7d

Study or Subgroup	High protein Mean	SD	Total	Low protein Mean	SD	Total	Weight	Mean Difference IV, Random, 95% CI
Bloomfield 2022	−0.94	0.93	180	−0.86	1.08	176	19.2%	−0.08 [−0.29, 0.13]
Moltu 2013b	0.63	0.65	22	−0.25	0.58	21	17.4%	0.88 [0.51, 1.25]
Morgan 2014a	−0.93	1.06	63	−1.32	1.18	63	17.1%	0.39 [−0.00, 0.78]
Salas 2022	−1.3	1.2	21	−1.5	1.4	25	12.1%	0.20 [−0.55, 0.95]
Shah 2016	−0.71	1.08	49	−0.65	1.07	50	16.7%	−0.06 [−0.48, 0.36]
Vlaardingerbroek 2013	0.6	1.1	47	−0.35	1.01	97	17.4%	0.95 [0.58, 1.32]
**Total (95% CI)**			**382**			**432**	**100.0%**	**0.38 [−0.03, 0.80]**

Heterogeneity: $Tau^2 = 0.22$; $Chi^2 = 36.75$, df = 5 (P < 0.00001); $I^2 = 86\%$
Test for overall effect: Z = 1.82 (P = 0.07)

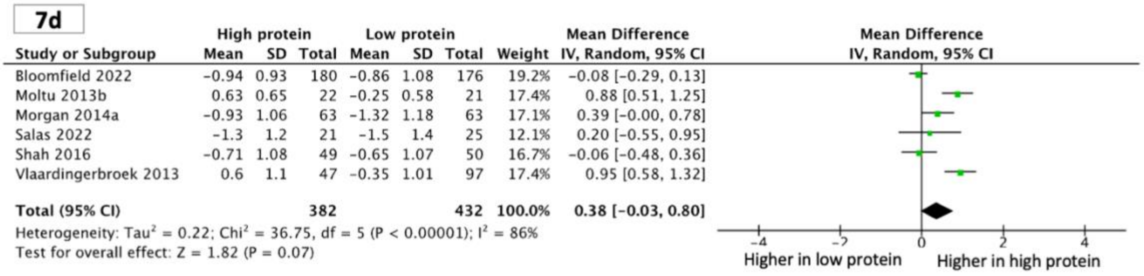

Supplementary Fig. 8: Forest plots of effects of planned high vs. low protein intake on a) hypophosphataemia, b) hypercalcaemia, c) refeeding syndrome, d) high blood urea, e) hyperglycaemia, f) hypoglycaemia, g) serum albumin level (g/dL) during infancy.

8a

Study or Subgroup	High protein Events	High protein Total	Low protein Events	Low protein Total	Weight	Risk Ratio M-H, Random, 95% CI
Bloomfield 2022	123	177	91	171	47.8%	1.31 [1.10, 1.55]
Lucas 1996	12	72	8	77	10.5%	1.60 [0.70, 3.70]
Moltu 2013a	17	22	6	23	13.2%	2.96 [1.44, 6.11]
Uthaya 2016	33	84	29	84	28.5%	1.14 [0.77, 1.69]
**Total (95% CI)**		**355**		**355**	**100.0%**	**1.43 [1.06, 1.93]**
Total events	185		134			

Heterogeneity: $Tau^2 = 0.04$; $Chi^2 = 5.57$, df = 3 (P = 0.13); $I^2 = 46\%$
Test for overall effect: Z = 2.33 (P = 0.02)

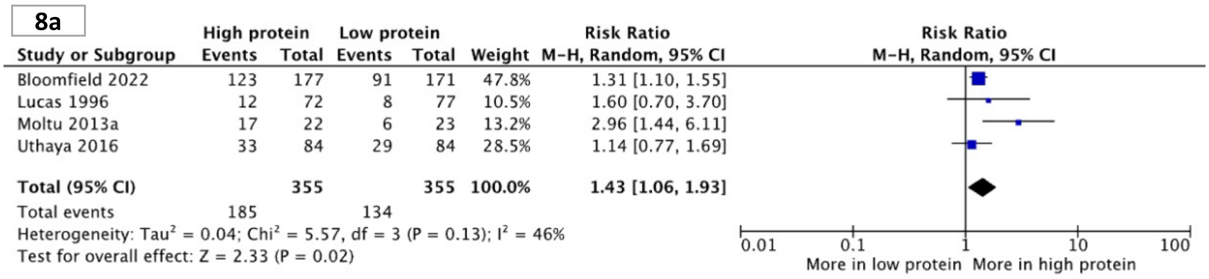

8b

Study or Subgroup	High protein Events	High protein Total	Low protein Events	Low protein Total	Weight	Odds Ratio M-H, Random, 95% CI
Bloomfield 2022	47	174	30	168	52.5%	1.70 [1.01, 2.86]
Lucas 1996	33	119	28	119	44.9%	1.25 [0.70, 2.24]
Uthaya 2016	5	84	0	84	2.6%	11.69 [0.64, 214.88]
**Total (95% CI)**		**377**		**371**	**100.0%**	**1.56 [0.97, 2.50]**
Total events	85		58			

Heterogeneity: $Tau^2 = 0.04$; $Chi^2 = 2.54$, df = 2 (P = 0.28); $I^2 = 21\%$
Test for overall effect: Z = 1.83 (P = 0.07)

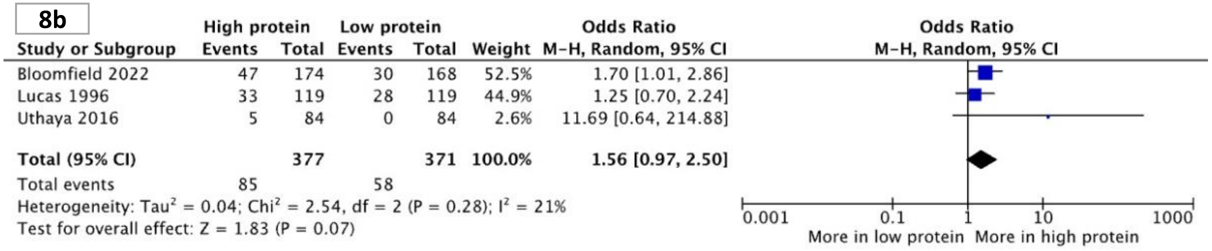

8c

Study or Subgroup	High protein Events	High protein Total	Low protein Events	Low protein Total	Weight	Risk Ratio M-H, Random, 95% CI
Bloomfield 2022	42	172	26	166	100.0%	1.56 [1.00, 2.42]
**Total (95% CI)**		**172**		**166**	**100.0%**	**1.56 [1.00, 2.42]**
Total events	42		26			

Heterogeneity: Not applicable
Test for overall effect: Z = 1.98 (P = 0.05)

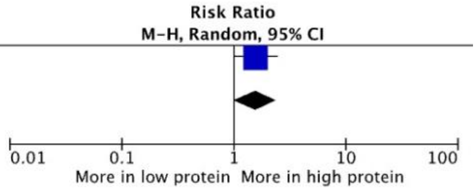

8d

Study or Subgroup	High protein Events	High protein Total	Low protein Events	Low protein Total	Weight	Risk Ratio M-H, Random, 95% CI
Lucas 1996	22	137	10	138	100.0%	2.22 [1.09, 4.50]
**Total (95% CI)**		**137**		**138**	**100.0%**	**2.22 [1.09, 4.50]**
Total events	22		10			

Heterogeneity: Not applicable
Test for overall effect: Z = 2.20 (P = 0.03)

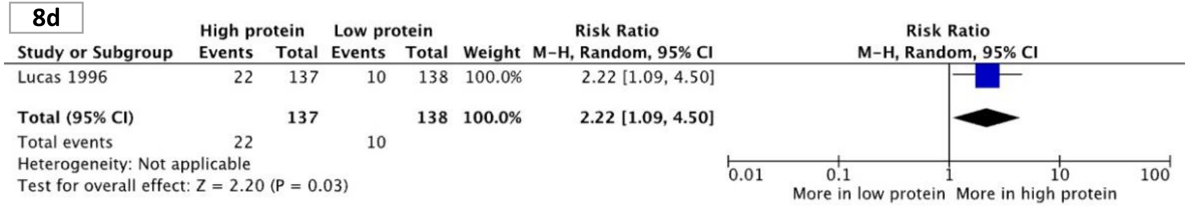

8e

Study or Subgroup	High protein Events	High protein Total	Low protein Events	Low protein Total	Weight	Risk Ratio M-H, Random, 95% CI
Burattini 2013	6	56	20	58	17.9%	0.31 [0.13, 0.72]
Tang 2009	8	34	18	62	22.3%	0.81 [0.39, 1.67]
Uthaya 2016	10	84	19	84	23.0%	0.53 [0.26, 1.06]
Wilson 1997	18	51	24	54	36.8%	0.79 [0.49, 1.28]
**Total (95% CI)**		**225**		**258**	**100.0%**	**0.61 [0.41, 0.92]**
Total events	42		81			

Heterogeneity: $Tau^2 = 0.06$; $Chi^2 = 4.50$, df = 3 (P = 0.21); $I^2 = 33\%$
Test for overall effect: Z = 2.36 (P = 0.02)

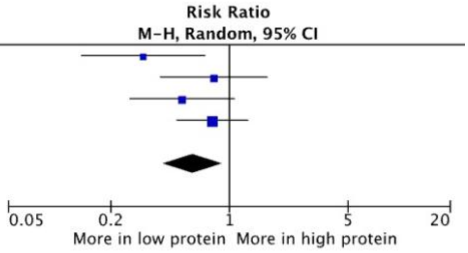

8f

Study or Subgroup	High protein Events	High protein Total	Low protein Events	Low protein Total	Weight	Risk Ratio M-H, Random, 95% CI
Uthaya 2016	31	84	31	84	100.0%	1.00 [0.67, 1.49]
**Total (95% CI)**		**84**		**84**	**100.0%**	**1.00 [0.67, 1.49]**
Total events	31		31			

Heterogeneity: Not applicable
Test for overall effect: Z = 0.00 (P = 1.00)

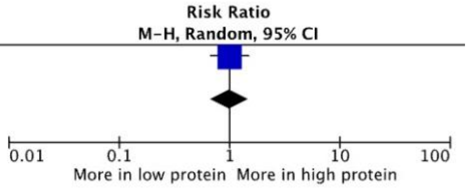

8g

Study or Subgroup	High protein Mean	High protein SD	High protein Total	Low protein Mean	Low protein SD	Low protein Total	Weight	Mean Difference IV, Random, 95% CI
Fairey 1997	3.4	0.3	8	3.1	0.2	7	14.6%	0.30 [0.04, 0.56]
Gupta 2020	2.9	0.4	71	2.8	0.4	67	20.8%	0.10 [−0.03, 0.23]
Hillman 1994	3.3	0.3	9	3.05	0.4	18	13.9%	0.25 [−0.02, 0.52]
Kashyap 1986	3.32	0.43	18	2.77	0.28	9	13.9%	0.55 [0.28, 0.82]
Moro 1991	3.8	0.37	9	3.9	0.31	12	12.6%	−0.10 [−0.40, 0.20]
Raiha 1996	3.55	0.11	41	3.2	0.17	65	24.1%	0.35 [0.30, 0.40]
**Total (95% CI)**			**156**			**178**	**100.0%**	**0.25 [0.10, 0.40]**

Heterogeneity: $Tau^2 = 0.02$; $Chi^2 = 22.13$, df = 5 (P = 0.0005); $I^2 = 77\%$
Test for overall effect: Z = 3.20 (P = 0.001)

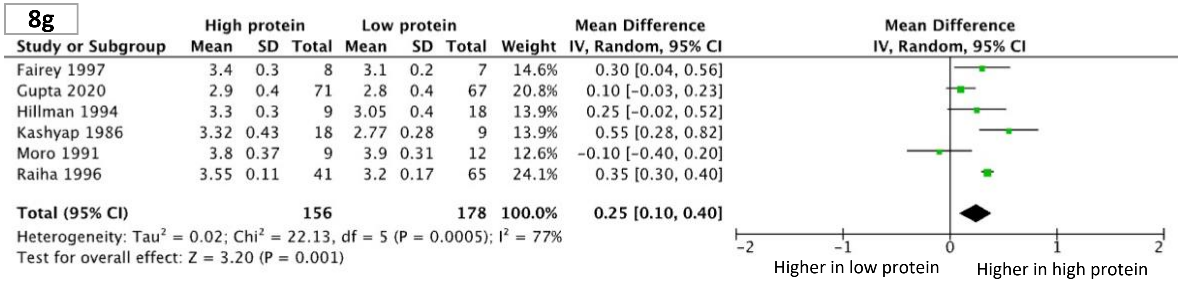

Supplementary Fig. 9: Forest plots of effects of planned high vs. low protein intake on a) fasting blood glucose (mg/dL) during infancy, b) lipid (triglyceride) concentration (mg/dl) during infancy, c) systolic blood pressure (mmHg) in childhood, and d) diastolic blood pressure (mmHg) in childhood.

9a

	High protein			Low protein				Mean Difference
Study or Subgroup	Mean	SD	Total	Mean	SD	Total	Weight	IV, Random, 95% CI
Balakrishnan 2018	122	48	85	119	36	83	16.9%	3.00 [-9.81, 15.81]
Ibrahim 2004	83.8	5.2	16	101.1	5.4	16	28.5%	-17.30 [-20.97, -13.63]
Schultz 1980	58.7	6.7	10	69.8	5.6	10	26.6%	-11.10 [-16.51, -5.69]
Tang 2009	101.3	34	34	119.47	42.44	62	13.9%	-18.17 [-33.73, -2.61]
Vlaardingerbroek 2013	113.4	46.8	47	107.12	37.67	97	14.1%	6.28 [-9.06, 21.62]
**Total (95% CI)**			**192**			**268**	**100.0%**	**-9.01 [-16.91, -1.12]**

Heterogeneity: Tau² = 53.45; Chi² = 17.94, df = 4 (P = 0.001); I² = 78%
Test for overall effect: Z = 2.24 (P = 0.03)

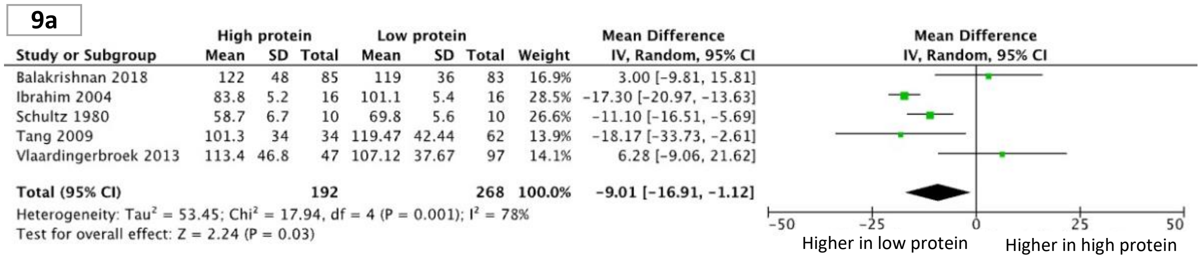

9b

	High protein			Low protein				Mean Difference
Study or Subgroup	Mean	SD	Total	Mean	SD	Total	Weight	IV, Random, 95% CI
Costa-Orvay 2011	74.75	21.1	24	106.5	58.9	8	12.3%	-31.75 [-73.43, 9.93]
Ibrahim 2004	70	9.8	16	84.9	10.1	16	43.9%	-14.90 [-21.80, -8.00]
Vlaardingerbroek 2013	36	21.6	47	32.4	17.04	97	43.8%	3.60 [-3.45, 10.65]
**Total (95% CI)**			**87**			**121**	**100.0%**	**-8.86 [-25.81, 8.09]**

Heterogeneity: Tau² = 157.80; Chi² = 14.99, df = 2 (P = 0.0006); I² = 87%
Test for overall effect: Z = 1.02 (P = 0.31)

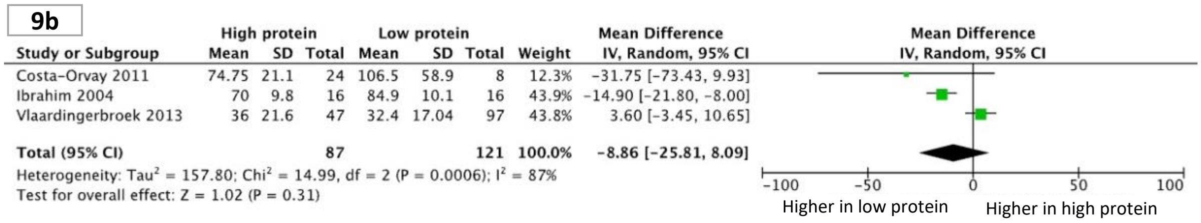

9c

	High protein			Low protein				Mean Difference
Study or Subgroup	Mean	SD	Total	Mean	SD	Total	Weight	IV, Random, 95% CI
Lucas 1989e	61.7	7.3	60	61.5	7.3	345	52.8%	0.20 [-1.80, 2.20]
Lucas 1990c	59.2	8	65	59.9	7.2	288	47.2%	-0.70 [-2.82, 1.42]
**Total (95% CI)**			**125**			**633**	**100.0%**	**-0.23 [-1.68, 1.23]**

Heterogeneity: Tau² = 0.00; Chi² = 0.37, df = 1 (P = 0.54); I² = 0%
Test for overall effect: Z = 0.30 (P = 0.76)

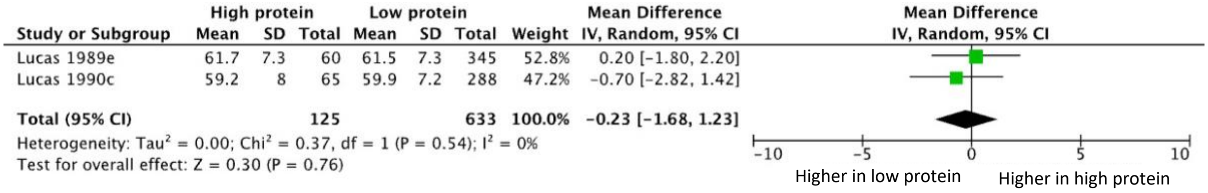

9d

	High protein			Low protein				Mean Difference
Study or Subgroup	Mean	SD	Total	Mean	SD	Total	Weight	IV, Random, 95% CI
Lucas 1989e	98.6	9.5	60	99.1	9.4	345	54.6%	-0.50 [-3.10, 2.10]
Lucas 1990c	101.8	10.8	65	101.9	9.7	288	45.4%	-0.10 [-2.95, 2.75]
**Total (95% CI)**			**125**			**633**	**100.0%**	**-0.32 [-2.24, 1.60]**

Heterogeneity: Tau² = 0.00; Chi² = 0.04, df = 1 (P = 0.84); I² = 0%
Test for overall effect: Z = 0.32 (P = 0.75)

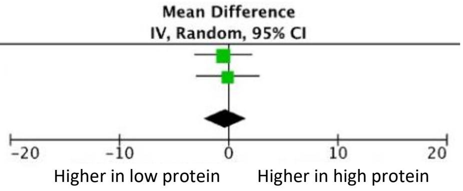

Supplementary Fig. 10: Forest plots of effects of planned high vs. low protein intake on a) survival without neurodisability at or beyond 12 months, b) length/height during the toddler period including only trials that achieved the planned intake.

10a

Study or Subgroup	High protein Events	High protein Total	Low protein Events	Low protein Total	Weight	Risk Ratio M-H, Random, 95% CI
Balakrishnan 2018	30	53	32	58	7.1%	1.03 [0.74, 1.43]
Bloomfield 2022	97	203	102	205	19.7%	0.96 [0.79, 1.17]
Lucas1989a	49	56	319	344	73.1%	0.94 [0.85, 1.05]
**Total (95% CI)**		**312**		**607**	**100.0%**	**0.95 [0.87, 1.04]**
Total events	176		453			

Heterogeneity: Tau² = 0.00; Chi² = 0.29, df = 2 (P = 0.87); I² = 0%

Test for overall effect: Z = 1.08 (P = 0.28)

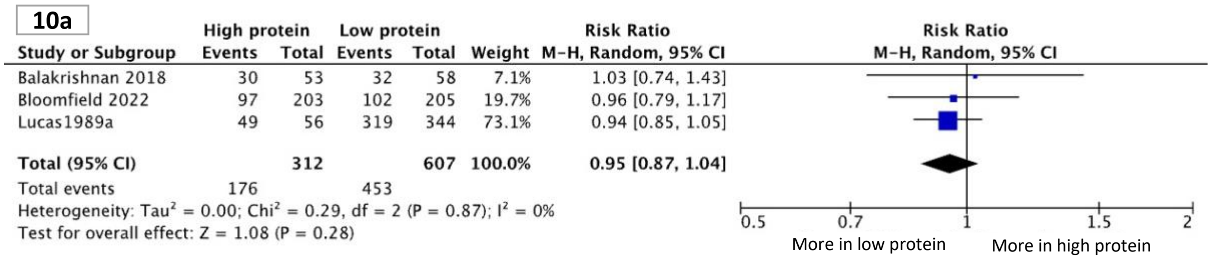

10b

Study or Subgroup	High protein Mean	High protein SD	High protein Total	Low protein Mean	Low protein SD	Low protein Total	Weight	Mean Difference IV, Random, 95% CI
Bellagamba 2016	86.8	3.4	82	87.4	4.4	82	15.5%	−0.60 [−1.80, 0.60]
Bloomfield 2022	85.9	3.5	155	86.7	4.2	157	23.8%	−0.80 [−1.66, 0.06]
Lucas 1989d	79.3	3.7	64	79.15	3.6	374	20.4%	0.15 [−0.83, 1.13]
Lucas 1990e	80.9	4.5	61	79.94	3.33	273	15.6%	0.96 [−0.24, 2.16]
Lucas 1996	80	3.35	125	80.1	3.29	120	24.6%	−0.10 [−0.93, 0.73]
**Total (95% CI)**			**487**			**1006**	**100.0%**	**−0.13 [−0.69, 0.43]**

Heterogeneity: Tau² = 0.15; Chi² = 6.43, df = 4 (P = 0.17); I² = 38%

Test for overall effect: Z = 0.44 (P = 0.66)

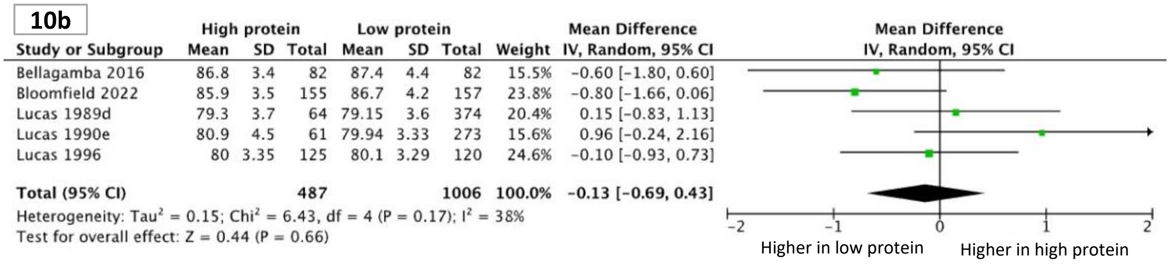

Supplementary Fig. 11: Forest plots of effects of planned high vs. low protein intake on a) survival without neurodisability at or beyond 12 months, b) survival to discharge or to 36 to 40 weeks, c) cognitive impairment or delay, d) motor impairment or delay, e) cerebral palsy during the toddler period and f) length (in cm) during the toddler period including trials with low risk of selection bias.

11a

Study or Subgroup	High protein Events	High protein Total	Low protein Events	Low protein Total	Weight	Risk Ratio M-H, Random, 95% CI
Balakrishnan 2018	30	53	32	58	7.1%	1.03 [0.74, 1.43]
Bloomfield 2022	97	203	102	205	19.7%	0.96 [0.79, 1.17]
Lucas1989a	49	56	319	344	73.1%	0.94 [0.85, 1.05]
**Total (95% CI)**		**312**		**607**	**100.0%**	**0.95 [0.87, 1.04]**
Total events	176		453			

Heterogeneity: Tau² = 0.00; Chi² = 0.29, df = 2 (P = 0.87); I² = 0%
Test for overall effect: Z = 1.08 (P = 0.28)

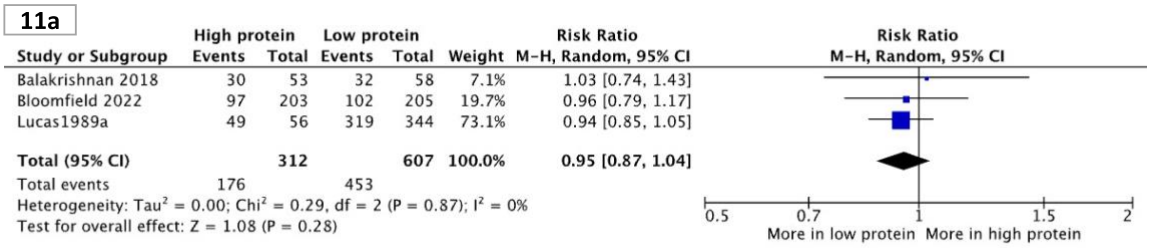

11b

Study or Subgroup	High protein Events	High protein Total	Low protein Events	Low protein Total	Weight	Risk Ratio M-H, Random, 95% CI
Blanco 2008a	26	30	27	31	3.1%	1.00 [0.82, 1.21]
Bloomfield 2022	179	217	178	217	15.7%	1.01 [0.92, 1.10]
Can 2012	25	26	25	27	6.9%	1.04 [0.91, 1.18]
Ibrahim 2004	14	16	15	16	2.4%	0.93 [0.75, 1.17]
Lucas 1996	130	137	129	138	34.6%	1.02 [0.96, 1.08]
Moltu 2013a	23	24	23	24	8.6%	1.00 [0.89, 1.13]
Morgan 2014a	63	74	64	76	6.4%	1.01 [0.88, 1.16]
Tan 2008a	55	68	59	74	4.5%	1.01 [0.86, 1.19]
Uthaya 2016	79	84	73	84	12.2%	1.08 [0.98, 1.19]
Vlaardingerbroek 2013	40	47	82	97	5.5%	1.01 [0.87, 1.17]
**Total (95% CI)**		**723**		**784**	**100.0%**	**1.02 [0.98, 1.05]**
Total events	634		675			

Heterogeneity: Tau² = 0.00; Chi² = 2.40, df = 9 (P = 0.98); I² = 0%
Test for overall effect: Z = 1.03 (P = 0.30)

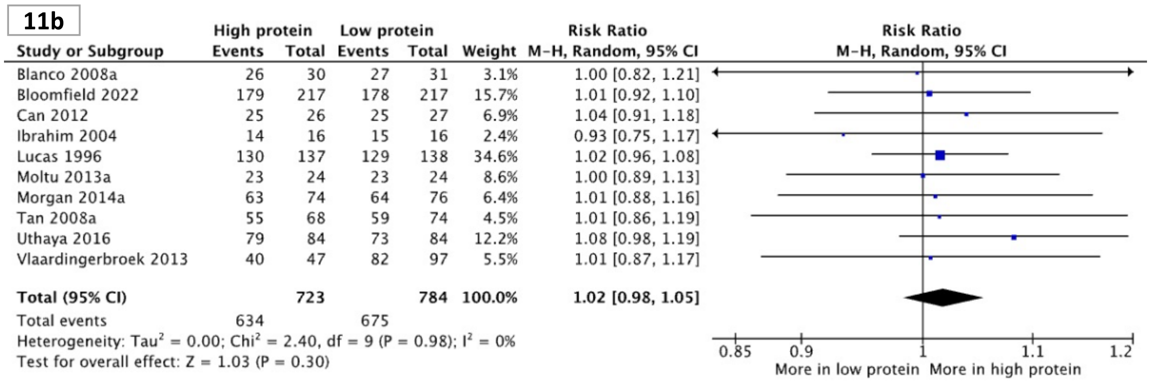

11c

Study or Subgroup	High protein Events	High protein Total	Low protein Events	Low protein Total	Weight	Risk Ratio M-H, Random, 95% CI
Bloomfield 2022	29	160	22	162	100.0%	1.33 [0.80, 2.22]
**Total (95% CI)**		**160**		**162**	**100.0%**	**1.33 [0.80, 2.22]**
Total events	29		22			

Heterogeneity: Not applicable
Test for overall effect: Z = 1.11 (P = 0.27)

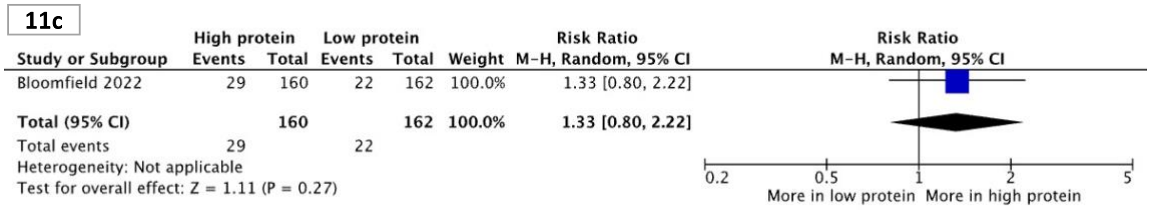

11d

Study or Subgroup	High protein Events	High protein Total	Low protein Events	Low protein Total	Weight	Risk Ratio M-H, Random, 95% CI
Bloomfield 2022	28	160	26	162	100.0%	1.09 [0.67, 1.77]
**Total (95% CI)**		**160**		**162**	**100.0%**	**1.09 [0.67, 1.77]**
Total events	28		26			

Heterogeneity: Not applicable
Test for overall effect: Z = 0.35 (P = 0.73)

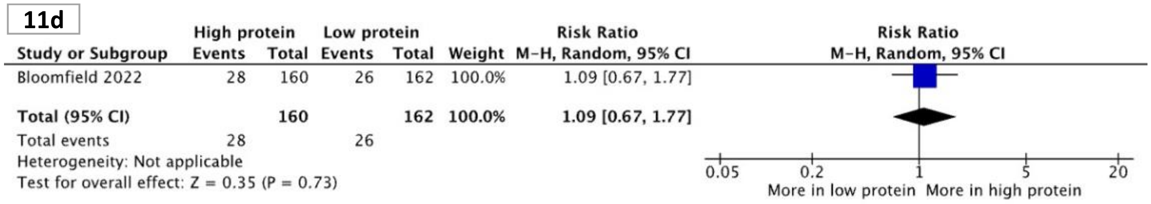

11e

Study or Subgroup	High protein Events	High protein Total	Low protein Events	Low protein Total	Weight	Risk Ratio M-H, Random, 95% CI
Blanco 2008a	3	16	1	16	7.5%	3.00 [0.35, 25.87]
Bloomfield 2022	13	161	9	163	33.8%	1.46 [0.64, 3.33]
Lucas 1989f	8	76	28	426	37.7%	1.60 [0.76, 3.38]
Lucas 1990a	1	81	15	343	8.5%	0.28 [0.04, 2.11]
Roelants 2018	5	45	2	89	12.6%	4.94 [1.00, 24.49]
**Total (95% CI)**		**379**		**1037**	**100.0%**	**1.62 [0.87, 3.00]**
Total events	30		55			

Heterogeneity: Tau² = 0.12; Chi² = 5.24, df = 4 (P = 0.26); I² = 24%
Test for overall effect: Z = 1.53 (P = 0.12)

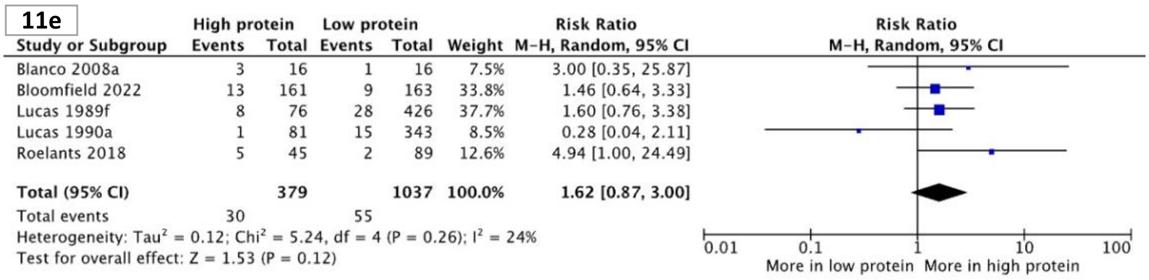

11f

Study or Subgroup	High protein Mean	High protein SD	High protein Total	Low protein Mean	Low protein SD	Low protein Total	Weight	Mean Difference IV, Random, 95% CI
Bloomfield 2022	85.9	3.5	155	86.7	4.2	157	27.8%	−0.80 [−1.66, 0.06]
Lucas 1989d	79.3	3.7	64	79.15	3.6	374	24.4%	0.15 [−0.83, 1.13]
Lucas 1990e	80.9	4.5	61	79.94	3.33	273	19.3%	0.96 [−0.24, 2.16]
Lucas 1996	80	3.35	125	80.1	3.29	120	28.6%	−0.10 [−0.93, 0.73]
**Total (95% CI)**			**405**			**924**	**100.0%**	**−0.03 [−0.69, 0.63]**

Heterogeneity: Tau² = 0.22; Chi² = 5.83, df = 3 (P = 0.12); I² = 48%
Test for overall effect: Z = 0.09 (P = 0.93)

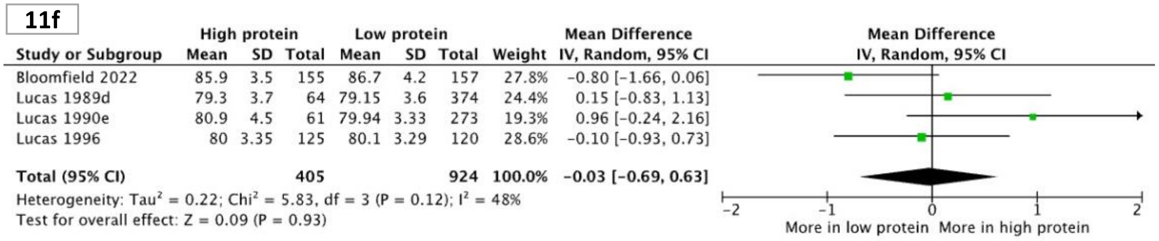

Supplementary Fig. 12: Forest plots of effects of planned high vs. low protein intake on a) survival to discharge or to 36 to 40 weeks, b) cerebral palsy during the toddler period, c) length during infancy, d) length during the toddler period including trials with low risk of detection bias.

12a

	High protein		Low protein			Risk Ratio
Study or Subgroup	Events	Total	Events	Total	Weight	M-H, Random, 95% CI
Balakrishnan 2018	76	85	73	83	20.4%	1.02 [0.91, 1.13]
Bloomfield 2022	179	217	178	217	31.3%	1.01 [0.92, 1.10]
Morgan 2014a	63	74	64	76	12.9%	1.01 [0.88, 1.16]
Uthaya 2016	79	84	73	84	24.4%	1.08 [0.98, 1.19]
Vlaardingerbroek 2013	40	47	82	97	11.1%	1.01 [0.87, 1.17]
**Total (95% CI)**		**507**		**557**	**100.0%**	**1.03 [0.98, 1.08]**
Total events	437		470			

Heterogeneity: Tau² = 0.00; Chi² = 1.51, df = 4 (P = 0.83); I² = 0%
Test for overall effect: Z = 1.06 (P = 0.29)

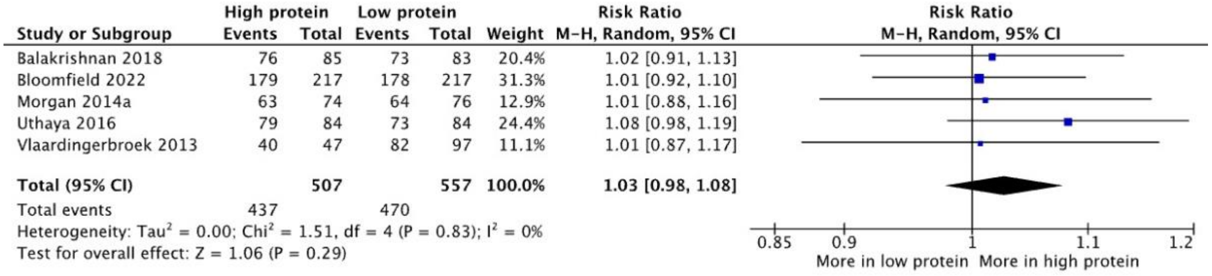

12b

	High protein		Low protein			Risk Ratio
Study or Subgroup	Events	Total	Events	Total	Weight	M-H, Random, 95% CI
Balakrishnan 2018	4	55	6	59	17.0%	0.72 [0.21, 2.40]
Bellagamba 2016	1	76	2	75	5.5%	0.49 [0.05, 5.33]
Bloomfield 2022	13	161	9	163	28.0%	1.46 [0.64, 3.33]
Lucas 1989f	8	76	28	426	30.9%	1.60 [0.76, 3.38]
Lucas 1990a	1	81	15	343	7.5%	0.28 [0.04, 2.11]
Roelants 2018	5	45	2	89	11.0%	4.94 [1.00, 24.49]
**Total (95% CI)**		**494**		**1155**	**100.0%**	**1.27 [0.71, 2.28]**
Total events	32		62			

Heterogeneity: Tau² = 0.14; Chi² = 6.93, df = 5 (P = 0.23); I² = 28%
Test for overall effect: Z = 0.79 (P = 0.43)

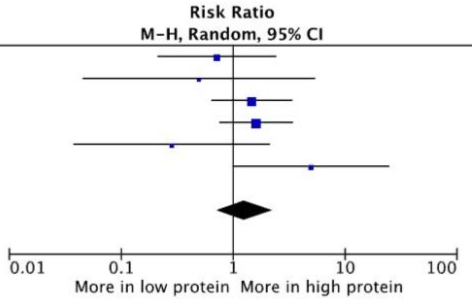

12c

	High protein			Low protein				Mean Difference
Study or Subgroup	Mean	SD	Total	Mean	SD	Total	Weight	IV, Random, 95% CI
Lucas 1989d	69.2	3.7	48	69.32	3.22	321	51.7%	-0.12 [-1.22, 0.98]
Lucas 1996	69.8	3.35	125	69.3	5.55	123	48.3%	0.50 [-0.64, 1.64]
**Total (95% CI)**			**173**			**444**	**100.0%**	**0.18 [-0.61, 0.97]**

Heterogeneity: Tau² = 0.00; Chi² = 0.58, df = 1 (P = 0.44); I² = 0%
Test for overall effect: Z = 0.44 (P = 0.66)

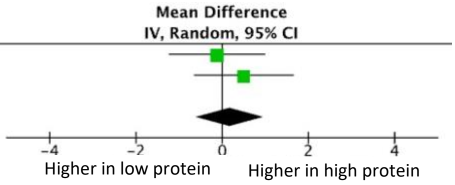

12d

	High protein			Low protein				Mean Difference
Study or Subgroup	Mean	SD	Total	Mean	SD	Total	Weight	IV, Random, 95% CI
Bellagamba 2016	86.8	3.4	82	87.4	4.4	82	18.6%	-0.60 [-1.80, 0.60]
Bloomfield 2022	85.9	3.5	155	86.7	4.2	157	27.5%	-0.80 [-1.66, 0.06]
Burattini 2013	87.3	4.6	48	87.4	4.1	52	11.1%	-0.10 [-1.81, 1.61]
Lucas 1989d	79.3	3.7	64	79.15	3.6	374	24.0%	0.15 [-0.83, 1.13]
Lucas 1990e	80.9	4.5	61	79.94	3.33	273	18.8%	0.96 [-0.24, 2.16]
**Total (95% CI)**			**410**			**938**	**100.0%**	**-0.13 [-0.77, 0.51]**

Heterogeneity: Tau² = 0.20; Chi² = 6.41, df = 4 (P = 0.17); I² = 38%
Test for overall effect: Z = 0.39 (P = 0.70)

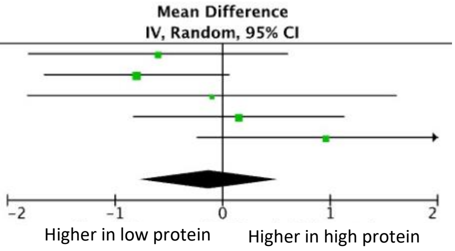

Supplementary Fig. 13. a) Risk of bias judgement: review authors' judgements about each risk of bias item presented percentages across all included study. b) Risk of bias summary: review authors' judgements about each risk of bias item for each included study.

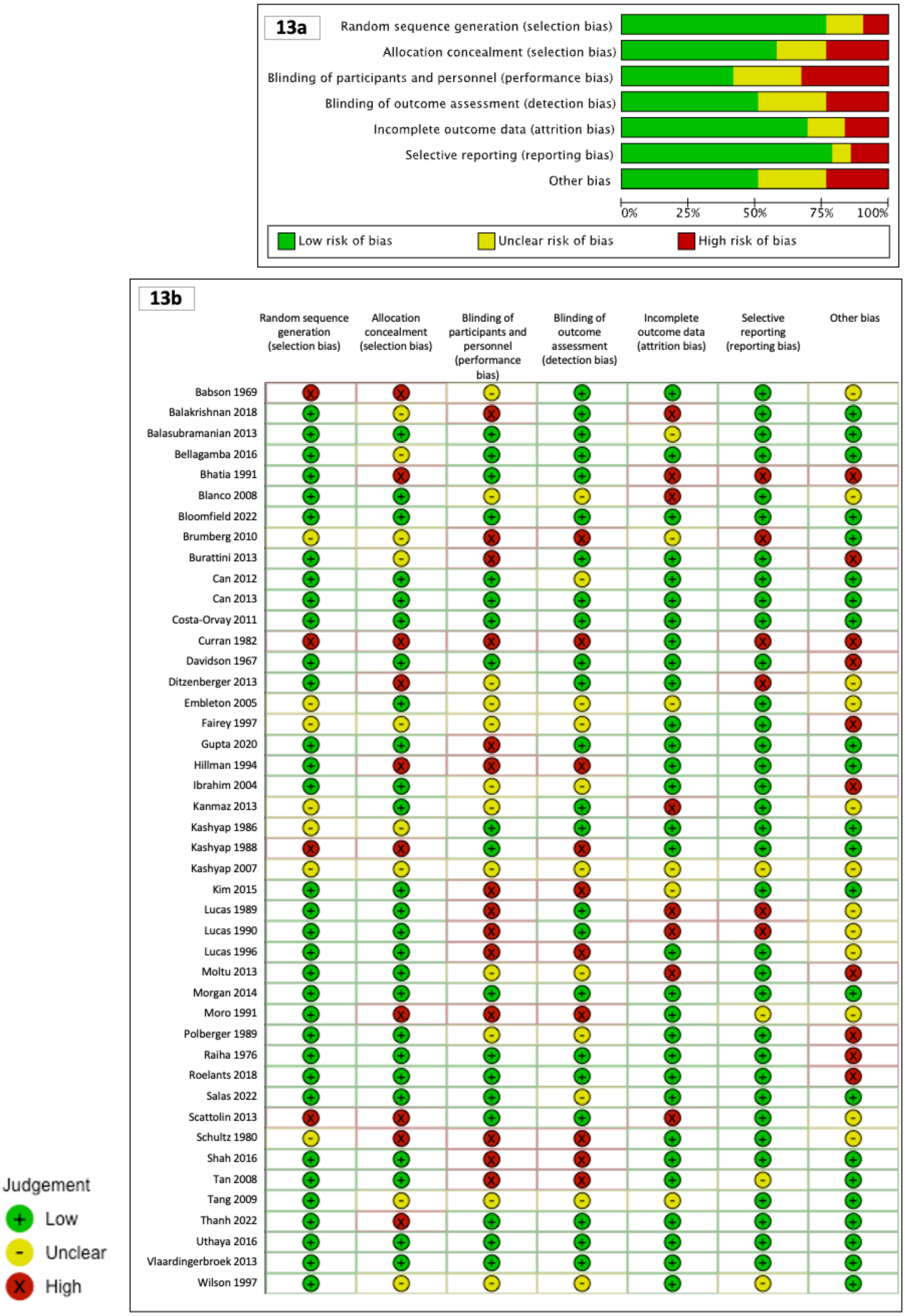

Supplementary Fig. 14. Risk of bias at outcome level assessed by ROB-2 tool. a. Risk of bias summary: review authors' judgements about each risk of bias item for each included study. Panel b. Risk of bias judgement: review authors' judgements about each risk of bias item presented as percentages across all included studies.

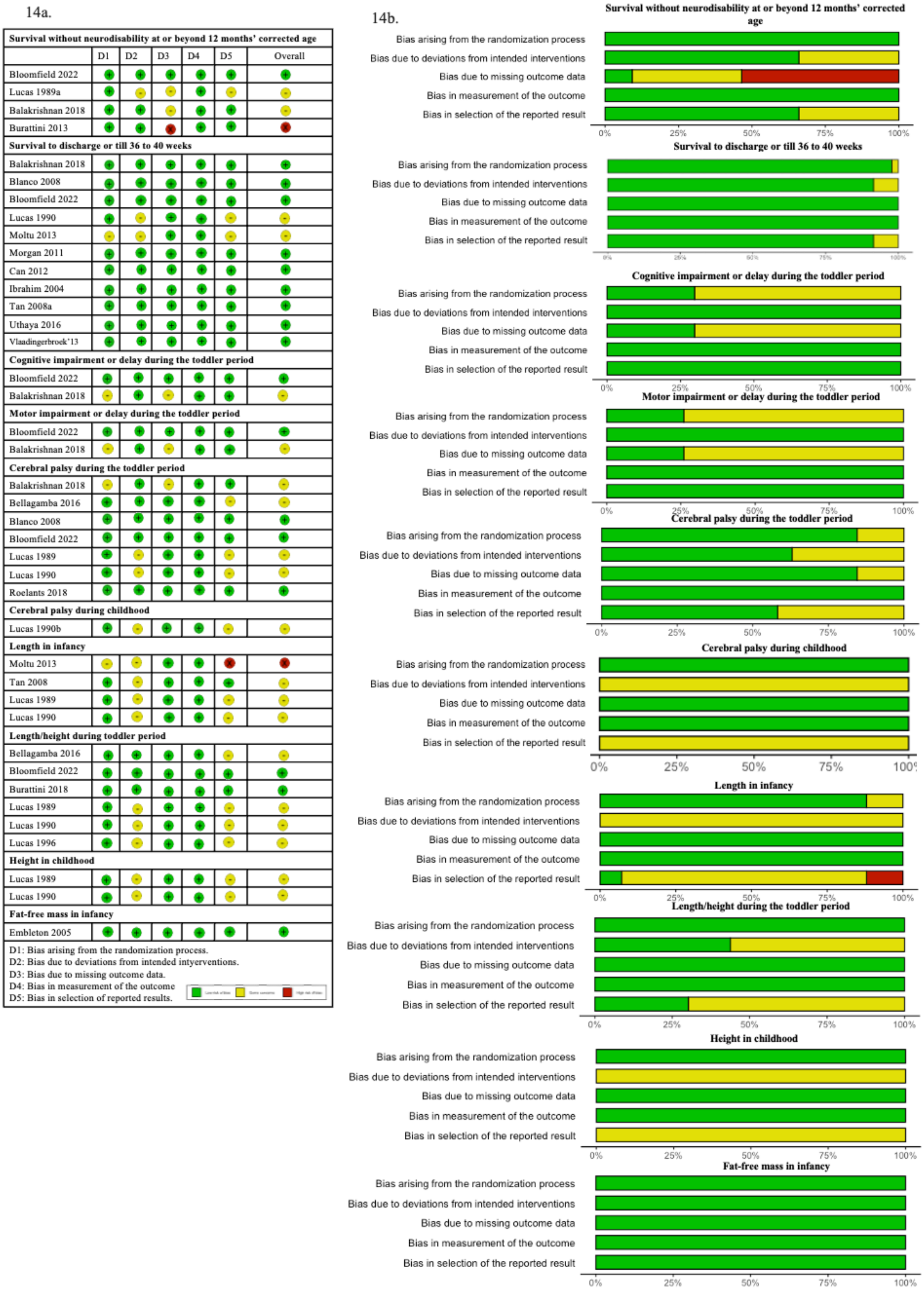

Supplement: Supplementary file 1 — SupplementaryFigures [file 41390_2024_3296_MOESM1_ESM.pdf]
