# Supplementary material for: High protein intake on later outcomes in preterm children: a systematic review and meta-analysis
Source: Pediatr Res. 2024 Jun 10;97(1):67–80. doi: 10.1038/s41390-024-03296-z (PMC11798874; doi:10.1038/s41390-024-03296-z)

Supplementary file 1. Funnel plots

A. Outcome: Survival to discharge or to 36 to 40 weeks

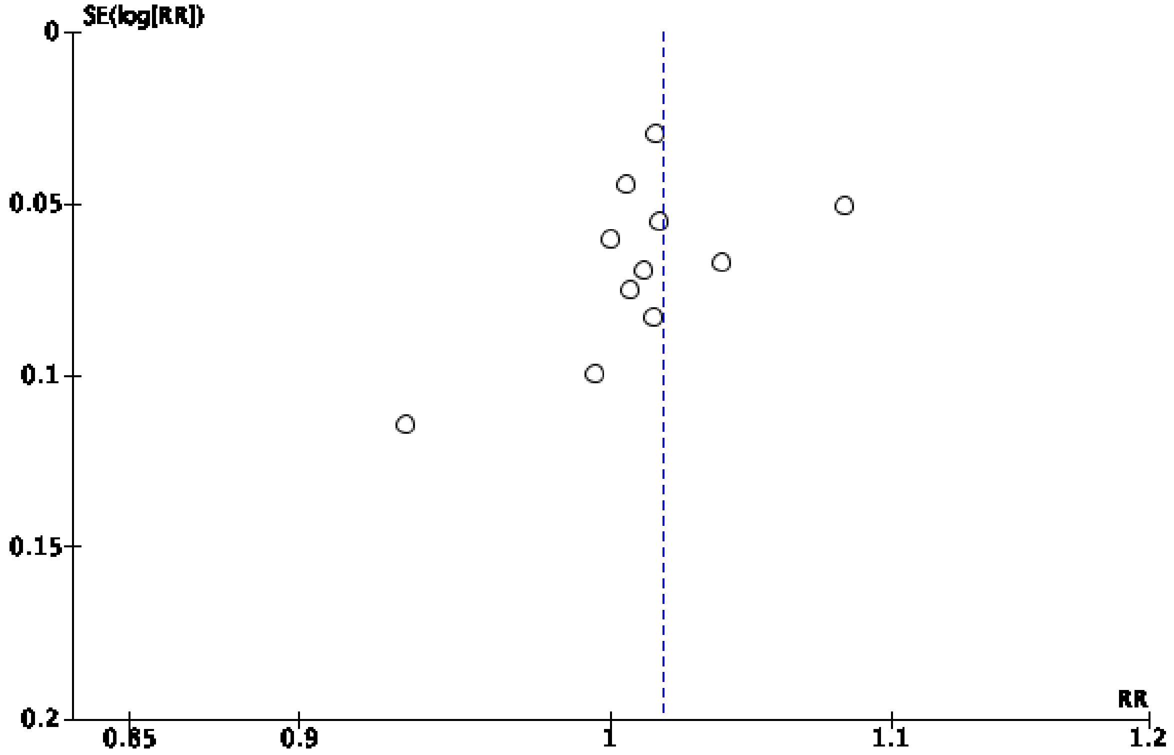

B. Outcome: Intraventricular haemorrhage

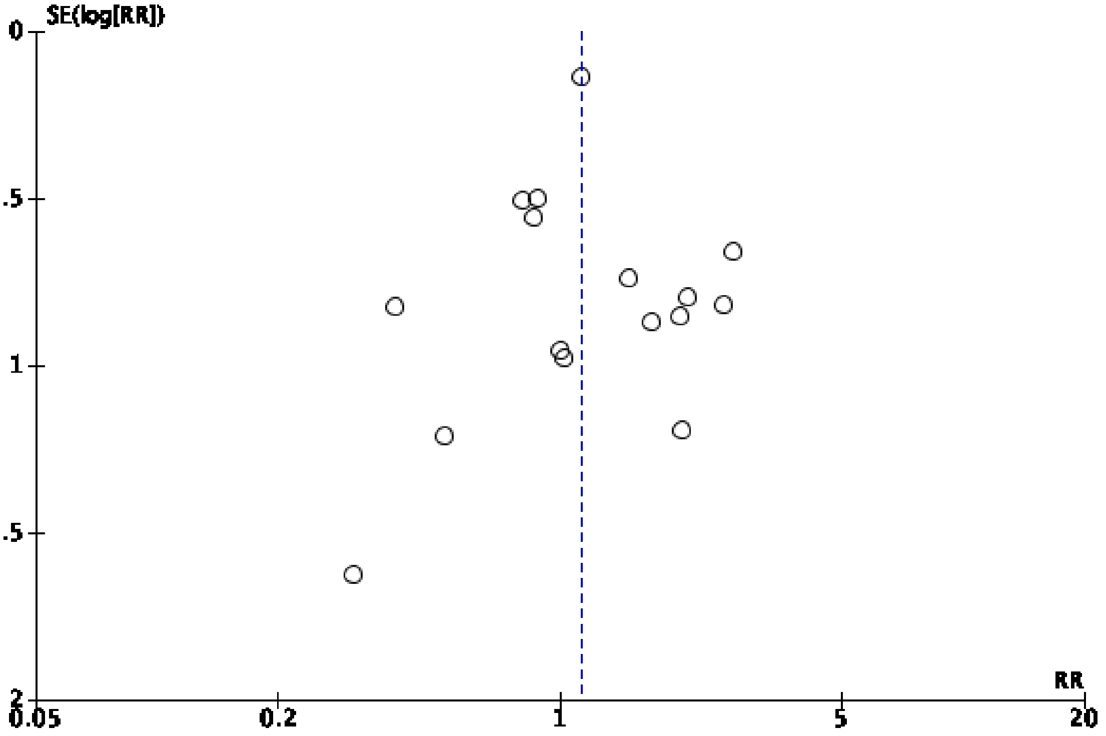

C. Outcome: Bronchopulmonary dysplasia

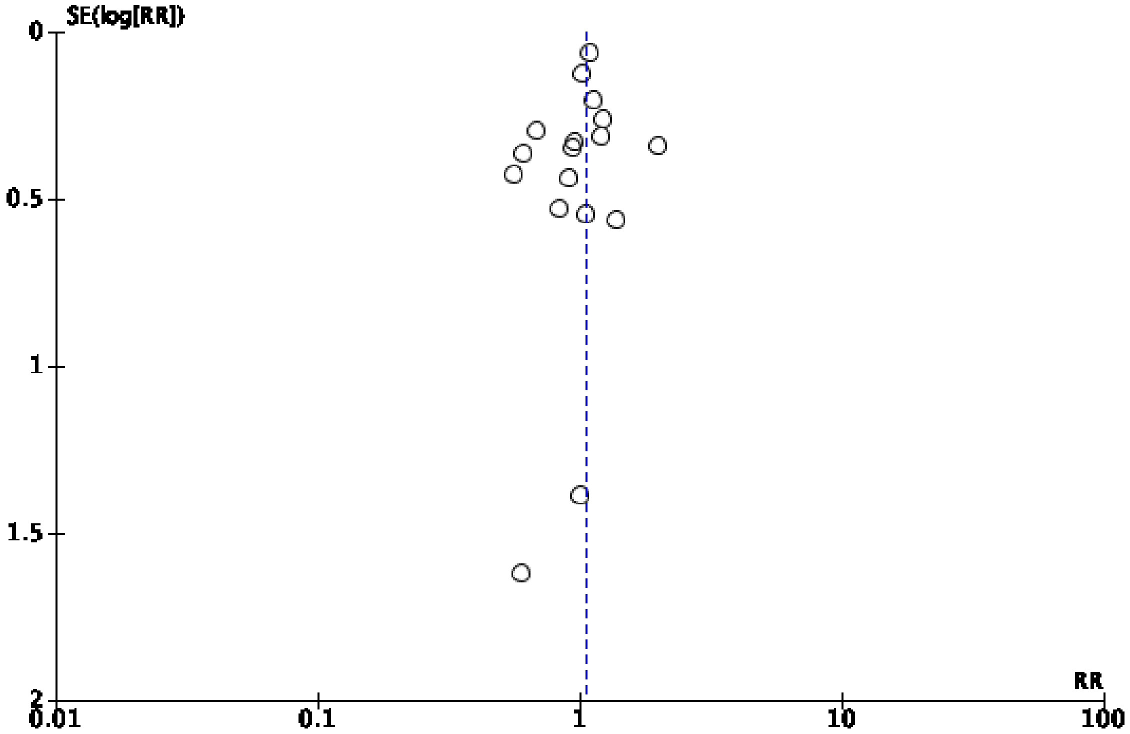

D. Outcome: Retinopathy of prematurity

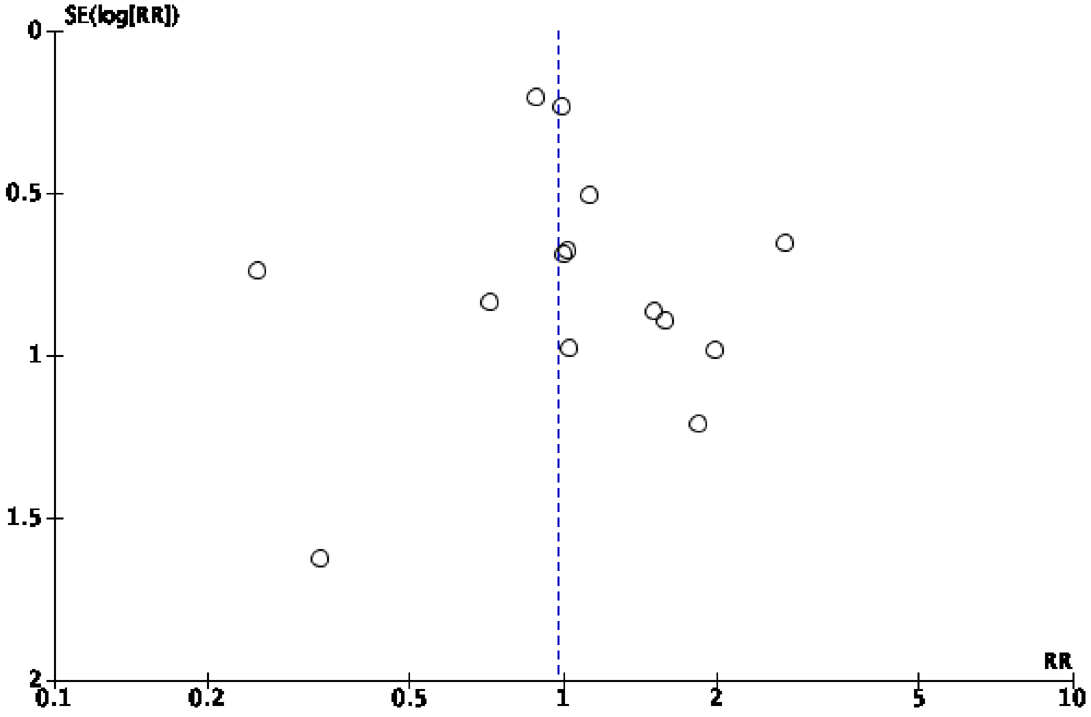

E. Outcome: Necrotising enterocolitis

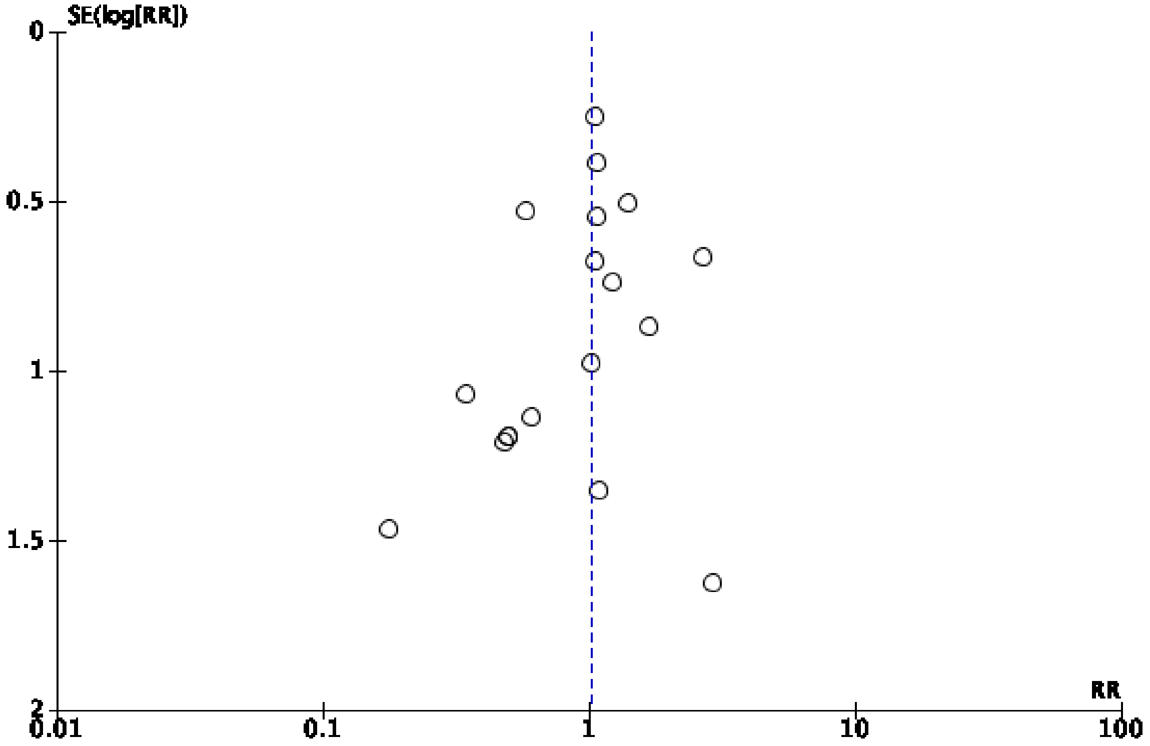

F. Outcome: Patent ductus arteriosus

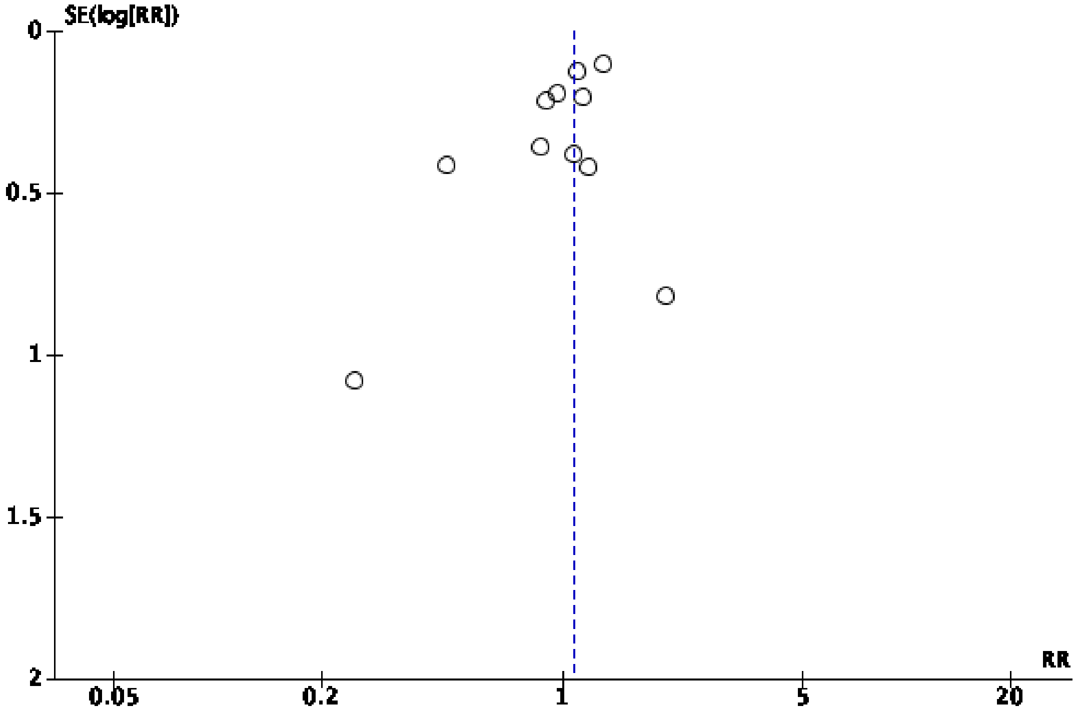

G. Outcome: Weight at discharge or at 36 weeks

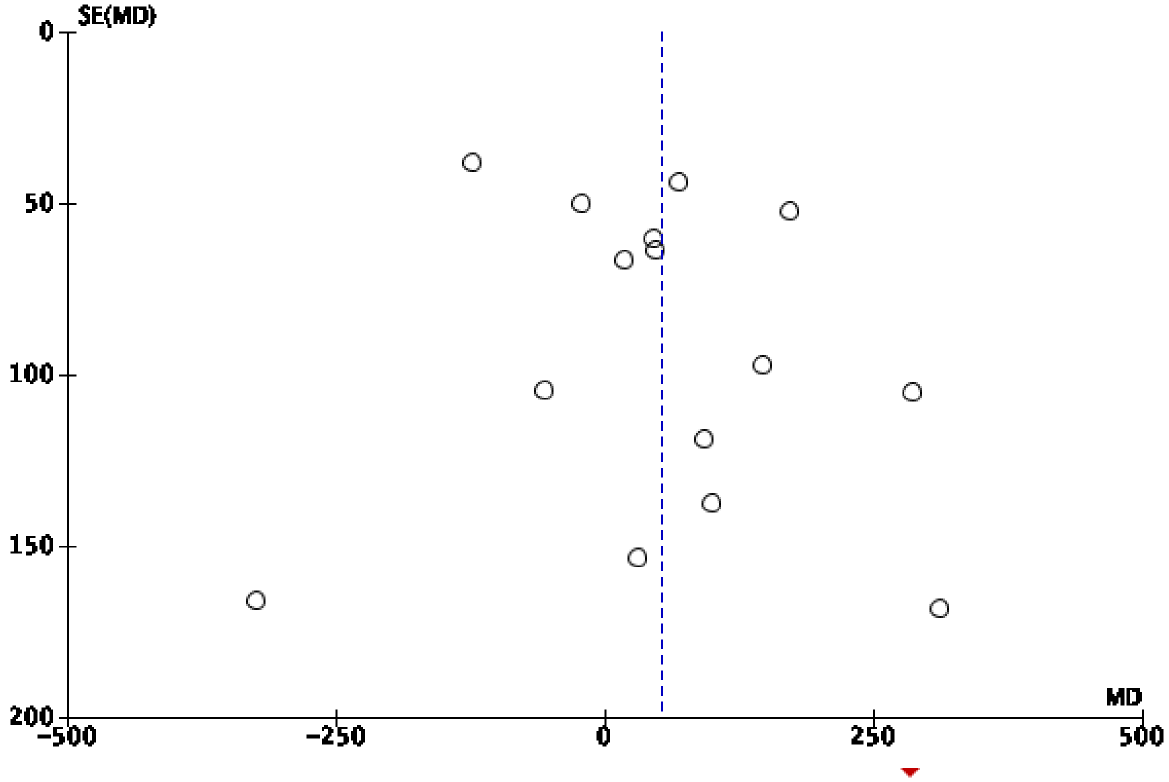

H. Outcome: Weight z-score at discharge or at 36 weeks

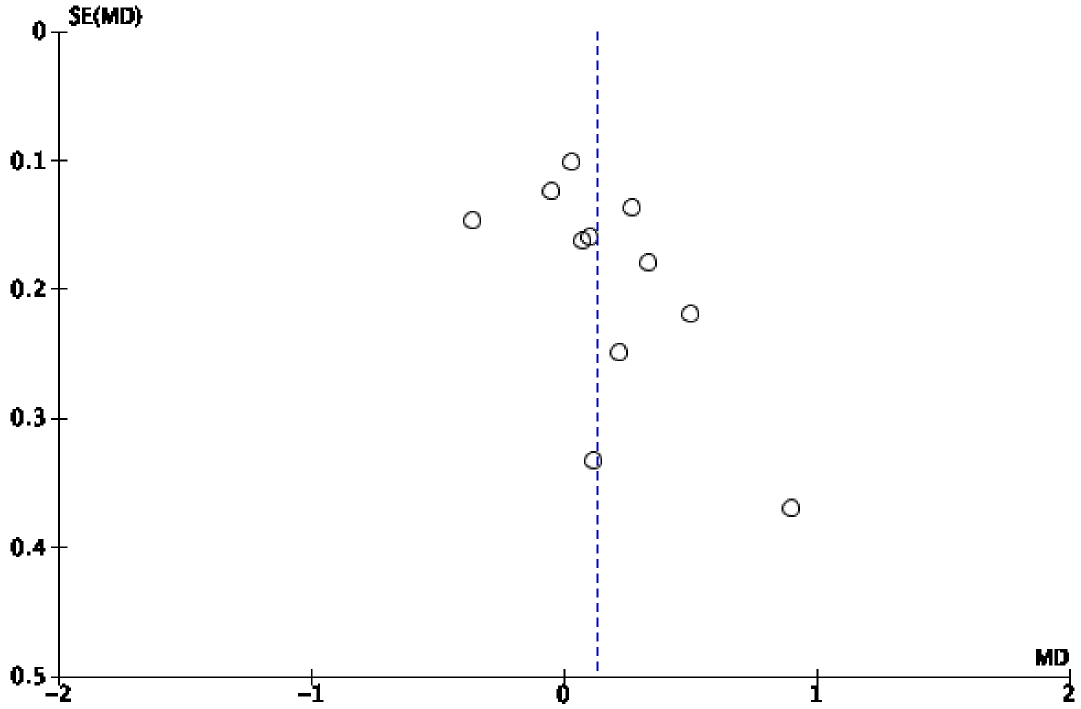

I. Outcome: Length at discharge or at 36 weeks

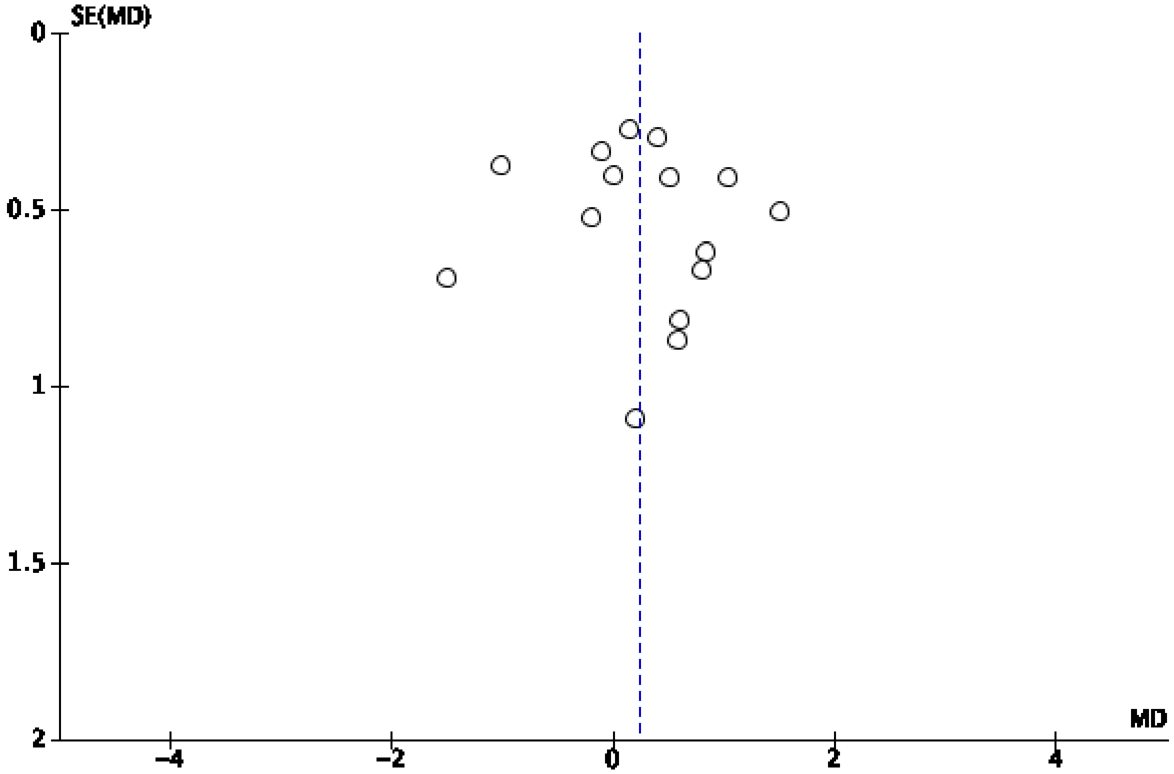

J. Outcome: Head circumference at discharge or at 36 weeks

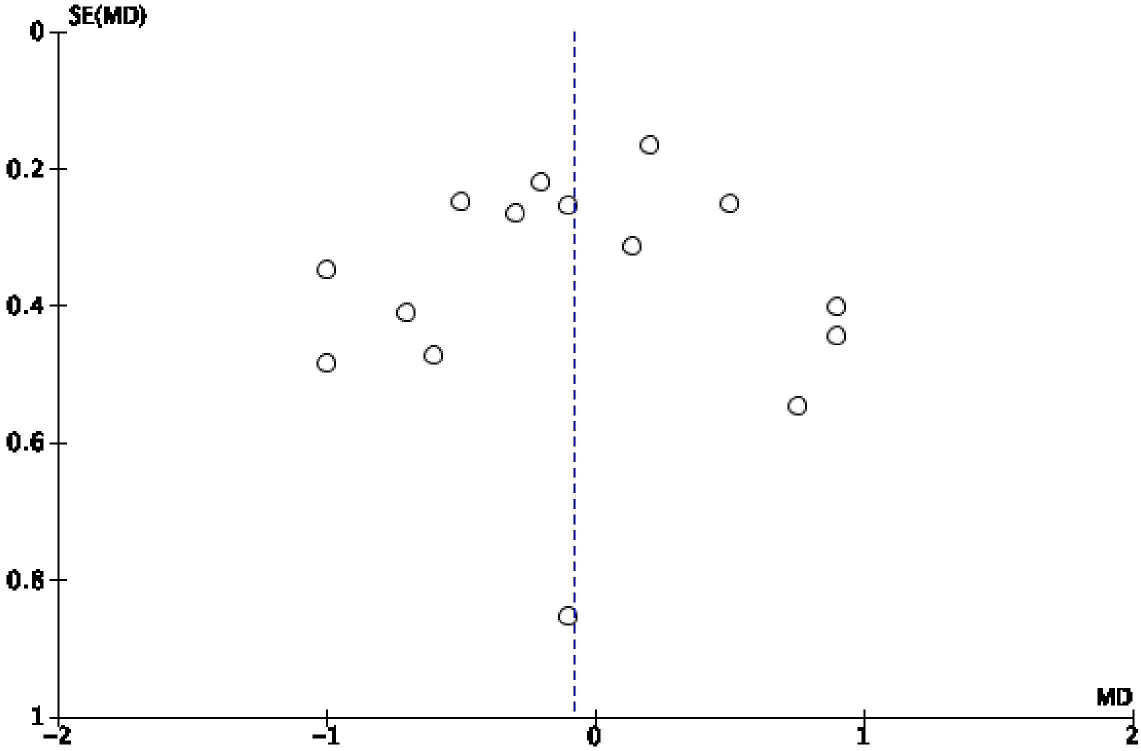

K. Outcome: Gain in head circumference till discharge or 36 weeks

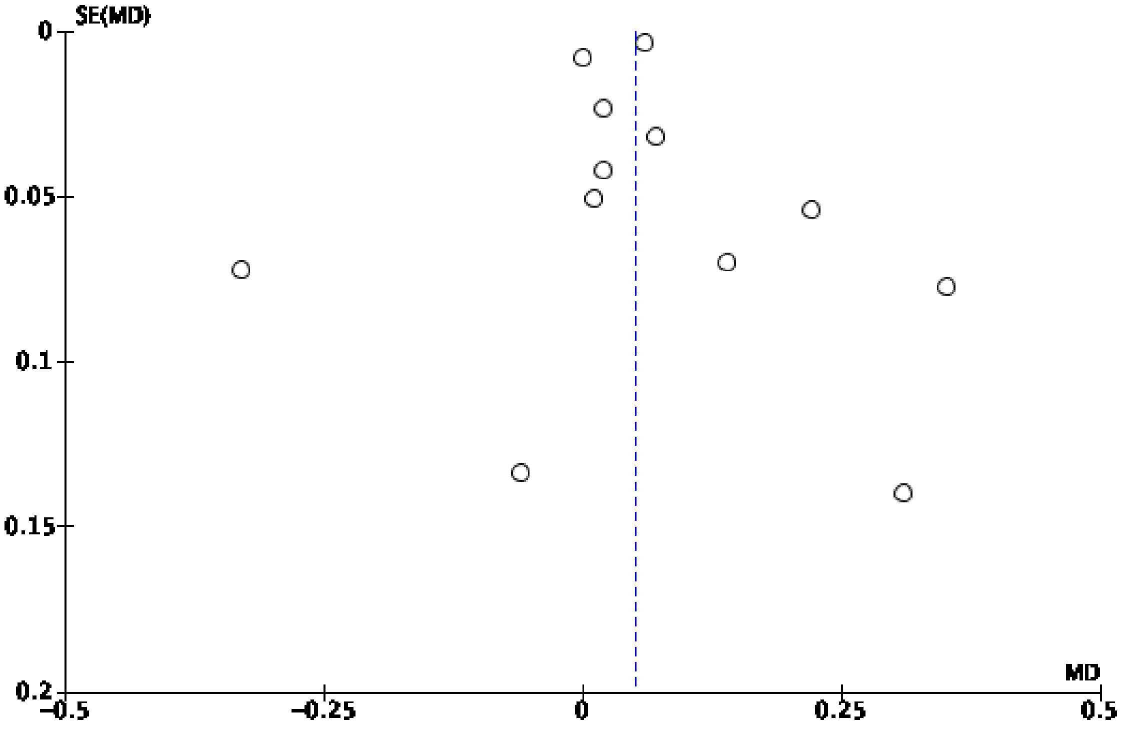

Supplement: Supplementary file 2 — Supplementary file 1 [file 41390_2024_3296_MOESM2_ESM.pdf]
