# Supplementary material for: Correction: High protein intake on later outcomes in preterm children: a systematic review and meta-analysis
Source: Pediatr Res. 2024 Nov 11;97(2):863–4. doi: 10.1038/s41390-024-03592-8 (PMC12015103; doi:10.1038/s41390-024-03592-8)
Supplement: Supplementary file 1 [file 41390_2024_3592_MOESM1_ESM.odt]

Incorrect Supplementary Figures 7b and 7c:

Ein Bild, das Text, Screenshot, Zahl, Schrift enthält.

Automatisch generierte Beschreibung

Corrected Supplementary Figures 7b and 7c:  

Ein Bild, das Text, Screenshot, Schrift, Zahl enthält.

Automatisch generierte Beschreibung
